# Emerging Multifunctional Carbon‐Nanomaterial‐Based Biosensors for Cancer Diagnosis

**DOI:** 10.1002/smsc.202300221

**Published:** 2024-01-22

**Authors:** Jolitta S. J. Britto, Xinwei Guan, Thi Kim Anh Tran, Zhihao Lei, Rohan Bahadur, Vaishwik Patel, Xiangwei Zhang, Sharon L. Wong, Ajayan Vinu

**Affiliations:** ^1^ Global Innovative Centre for Advanced Nanomaterials (GICAN) College of Engineering, Science and Environment (CESE) The University of Newcastle Callaghan NSW 2308 Australia; ^2^ Present address: STEM School of Science RMIT University Melbourne VIC 3001 Australia

**Keywords:** biomarkers, biosensors, cancer diagnosis, carbon nanomaterials, detection

## Abstract

Despite significant advancements in medical technology, cancer remains the world's second‐leading cause of death, largely attributed to late‐stage diagnoses. While traditional cancer detection methodologies offer foundational insights, they often lack the specificity, affordability, and sensitivity for early‐stage identification. In this context, the development of biosensors offers a distinct possibility for the precise and rapid identification of cancer biomarkers. Carbon nanomaterials, including graphene, carbon nitride, carbon quantum dots, and other carbon‐based nanostructures, are highly promising for cancer detection. Their simplicity, high sensitivity, and cost‐effectiveness contribute to their potential in this field. This review aims to elucidate the potential of emerging carbon‐nanomaterial‐based biosensors for early cancer diagnosis. The relevance of the various biosensor mechanisms and their performance to the physicochemical properties of carbon nanomaterials is discussed in depth, focusing on demonstrating broad methodologies for creating performance biosensors. Diverse carbon‐nanomaterial‐based detection techniques, such as electrochemical, fluorescence, surface plasmon resonance, electrochemiluminescence, and quartz crystal microbalance, are emphasized for early cancer detection. At last, a summary of existing challenges and future outlook in this promising field is elaborated.

## Graphical abstract

Carbon nanomaterials, including graphene, carbon nitride, and carbon quantum dots, are among the most promising building blocks as emerging biosensors for cancer detection. This review elaborates on the virtues and critical roles of carbon nanomaterials and summarizes the prevalent approaches for realizing high‐sensitive biosensor applications.

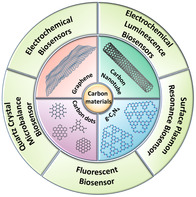

## 1 Introduction

According to the World Health Organization, cancer refers to a wide range of diseases that may originate in any organ or tissue of the body. These diseases are characterized by the uncontrolled growth of abnormal cells that can expand their normal boundaries, invade nearby tissues, and potentially spread to other organs.^[^ ^1^, ^2^, ^3^, ^4^ ^]^ The nature of cancer is the rapid proliferation of abnormal cells with the ability to spread to other organs,^[^ ^5^, ^6^, ^7^ ^]^ and its development generally follows several steps. First, normal genes in cells are mutated and become cancer‐related genes or oncogenes; this process is also known as proto‐oncogenesis. The formation of oncogenes through mutation or replication of normal genes is known as the activation of oncogenes. Subsequently, these activated oncogenes increase cell division, cell growth deregulation, and cancer formation, while tumor suppressor genes are downregulated. Ultimately, the mutated tumor suppressor genes are regulated for cell division and against programmed cell death (apoptosis), leading to eventual cancer.^[^ ^8^, ^9^, ^10^ ^]^

Cancer has rapidly emerged as the second most prominent cause of human mortality in modern society.^[^ ^11^, ^12^, ^13^, ^14^ ^]^ In the year 2020, there were 19.3 million new cancer cases diagnosed and over 10 million recorded cancer‐related deaths worldwide. Among these instances, female breast cancer constituted the most frequently diagnosed type at approximately 2.3 million (11.7%), followed by lung cancer (11.4%), colorectal (10.0%), prostate (7.3%), and stomach (5.6%) cancers. For cancer‐related deaths, lung cancer maintained its position as the primary cause with an estimated ≈1.8 million deaths (18%), followed by colorectal (9.4%), liver (8.3%), stomach (7.7%), and female breast (6.9%) cancers.^[^ ^15^, ^16^, ^17^ ^]^ Despite recent medical and technological advancements, cancer morbidity and mortality rates remain high, mainly due to late‐stage diagnoses and poor prognosis. In this regard, early cancer detection becomes a life‐saving support for patients to accelerate various treatment processes including nanodrug delivery.^[^ ^4^, ^17^, ^18^, ^19^, ^20^, ^21^, ^22^, ^23^ ^]^ A sensitive and accurate cancer diagnosis is a highly efficient approach for enhancing their chances of survival, particularly in developing countries.^[^ ^24^, ^25^, ^26^ ^]^ Conventional cancer diagnosis methods typically employ noninvasive nuclear‐based imaging techniques, including magnetic resonance imaging (MRI), computed tomography (CT), ultrasonography, positron emission tomography (PET), and single‐photon emission computed tomography. Moreover, invasive biopsy and histopathological examination are employed to identify cancer types and determine their stages.^[^ ^27^ ^]^ Although these methods have provided the foundation for cancer diagnosis and treatment, they are nonspecific, costly, and heavily dependent on the growth rate of cancer tumors.^[^ ^27^ ^]^ In addition, a wide variety of techniques within the field of molecular biology, such as enzyme‐linked immunosorbent assay,^[^ ^28^ ^]^ radioimmunoassay,^[^ ^29^ ^]^ immunohistochemistry,^[^ ^30^, ^31^ ^]^ flow cytometry,^[^ ^32^ ^]^ and DNA/RNA‐based hybridization/sequencing approaches,^[^ ^33^, ^34^, ^35^, ^36^ ^]^ have also been widely used to detect molecular signatures/biomarkers in cancer cells. Although current molecular diagnostic techniques have been clinically validated, they frequently exhibit low sensitivity, extended detection times, potential health risks, and the requirement for sophisticated equipment and skilled operators. These limitations significantly hinder their practicality and effectiveness in efficiently identifying low‐concentration biomarkers in early‐stage disease diagnosis.^[^ ^37^, ^38^, ^39^, ^40^, ^41^ ^]^ Therefore, developing novel detection approaches with efficient nanomaterials that are simple, sensitive, and rapid for early‐stage cancer diagnosis is of enormous clinical significance.

In the field of biosensing, the choice of materials becomes crucial not only for their intrinsic properties but also for their adaptability to interface with various biomolecules.^[^ ^42^ ^]^ To this end, carbon nanomaterials have a distinct edge: their carbonaceous nature lends itself to a plethora of functional groups that can be introduced either during synthesis or through post‐synthetic modifications. Such inherent versatility allows for conjugating a diverse range of biomolecules directly onto the carbon‐based scaffold. Unlike materials like gold, which predominantly rely on thiol chemistry,^[^ ^43^ ^]^ or silicon dioxide, which necessitates the use of silane groups,^[^ ^42^, ^44^ ^]^ carbon‐based nanomaterials can be easily functionalized using carboxyl, amine, hydroxyl, and other groups.^[^ ^45^, ^46^, ^47^, ^48^, ^49^, ^50^, ^51^ ^]^ Furthermore, the diverse allotropes of carbon, from graphene to carbon nanotubes, offer varying configurations and densities of these functional sites,^[^ ^52^, ^53^ ^]^ ensuring that a suitable carbon nanomaterial with the optimal density and orientation of functional groups can be chosen based on the specific requirements of the biosensing applications.

**Figure 1:**
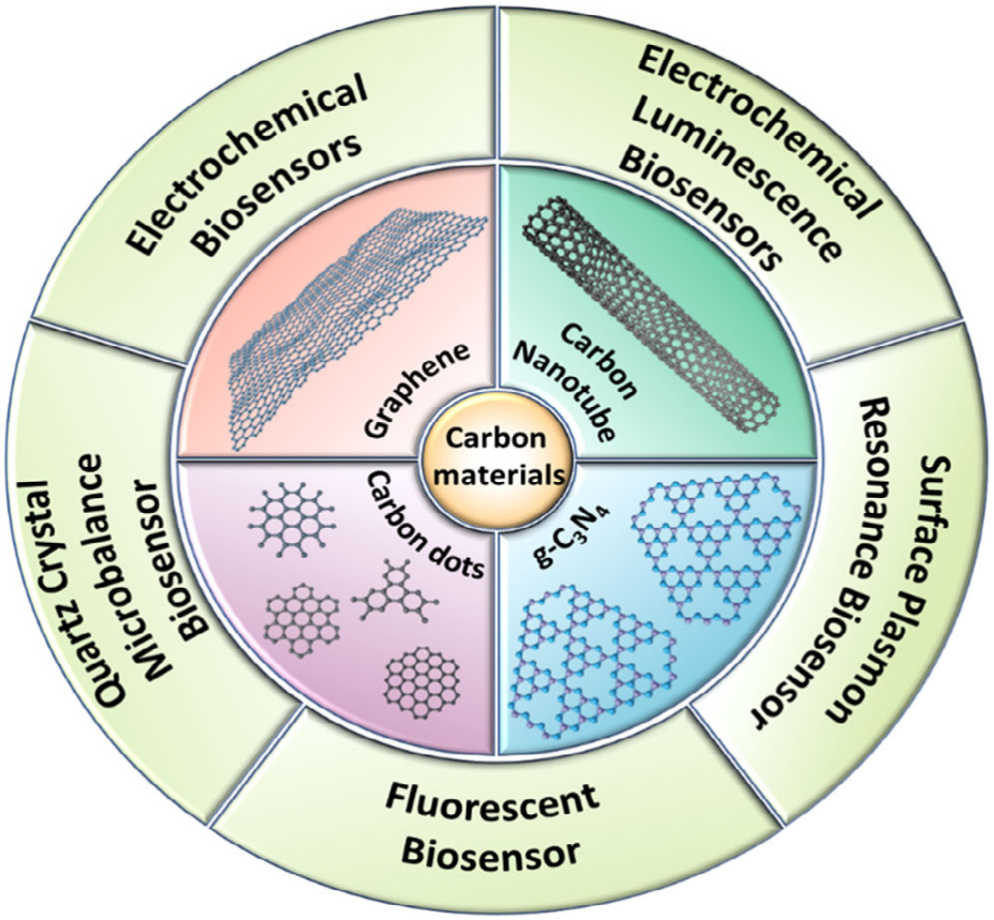
Different types of carbon nanomaterial biosensors based on their transducer operation.

To date, biosensor development is becoming a prevalent approach for effectual point‐of‐care testing with high‐level accuracy, reproducibility, reliability, stability, affordability, and disposability.^[^ ^54^, ^55^ ^]^ In particular, biosensor research is deemed as a required field to detect the toxins and cancer cells in the blood, and numerous researchers have developed biosensors to sense the different types of biomarkers responsible for cancer and other diseases.^[^ ^56^ ^]^ The recent development of biosensors has focused on five types of advanced sensors using different transducer elements to sense biomolecules, i.e., electrochemical,^[^ ^57^, ^58^ ^]^ fluorescence,^[^ ^59^ ^]^ electrochemiluminescence (ECL),^[^ ^60^ ^]^ surface plasmon resonance (SPR),^[^ ^61^ ^]^ and quartz crystal microbalance (QCM),^[^ ^62^ ^]^ which in turn produce measurable current, light, and frequency signals, enabling monitoring of analytes in extremely low concentrations (**Figure** **1**).^[^ ^63^, ^64^, ^65^ ^]^ Compared with the aforementioned analysis approaches, emerging nanomaterial‐based sensing devices are highly efficient and cost‐effective.^[^ ^66^, ^67^, ^68^ ^]^ In this context, carbon nanomaterials have drawn tremendous attention in medical science thanks to their outstanding electrical conductivity, affordable cost, large specific surface area, and biocompatibility.^[^ ^69^, ^70^, ^71^, ^72^, ^73^ ^]^ This ease and compatibility of functionalization, combined with their intrinsic properties, make carbon nanomaterials uniquely suited for biosensing applications.^[^ ^74^ ^]^ The high tunability of the surface chemistry for carbon nanomaterials enables them to be specific with target biomolecules, further enhancing their sensitivity and selectivity as diagnostic tools.^[^ ^75^, ^76^ ^]^ Carbon‐based nanostructures have been extensively employed in biosensors and therapy fields,^[^ ^77^, ^78^, ^79^ ^]^ which are essential for food, agriculture, and environmental monitoring applications.^[^ ^80^, ^81^ ^]^ Notably, carbon‐nanomaterial‐based biosensors indicate considerable high sensitivity, enabling a low‐concentration detection of the analyte molecules down to the picogram and femtogram levels.^[^ ^82^, ^83^, ^84^, ^85^ ^]^ Thereby, carbon‐based biosensors play an essential role in healthcare fields nowadays, especially in cancer research, where they are exploited to detect the concentration of the multiple targets/biomarkers responsible for particular cancer diseases. Among the carbon nanomaterials, graphene‐based biosensors attract the most attention owing to the unique crystalline structure with one single element and the advantages of low cost and high accuracy.^[^ ^86^, ^87^, ^88^ ^]^ In addition, carbon quantum dots (QDs) emerge as novel zero‐dimensional (0D) materials in sensing technology because of their high affinity, good intracellular solubility, and specificity for detecting cancer cells with higher accuracy.^[^ ^89^, ^90^, ^91^ ^]^ Besides, other carbon nanomaterials, such as porous carbon,^[^ ^92^ ^]^ graphic carbon nitride (g‐C_3_N_4_),^[^ ^93^, ^94^ ^]^ fullerene,^[^ ^95^ ^]^ and carbon nanotubes,^[^ ^85^ ^]^ also present a massive potential for biosensing applications.

**Figure 2:**
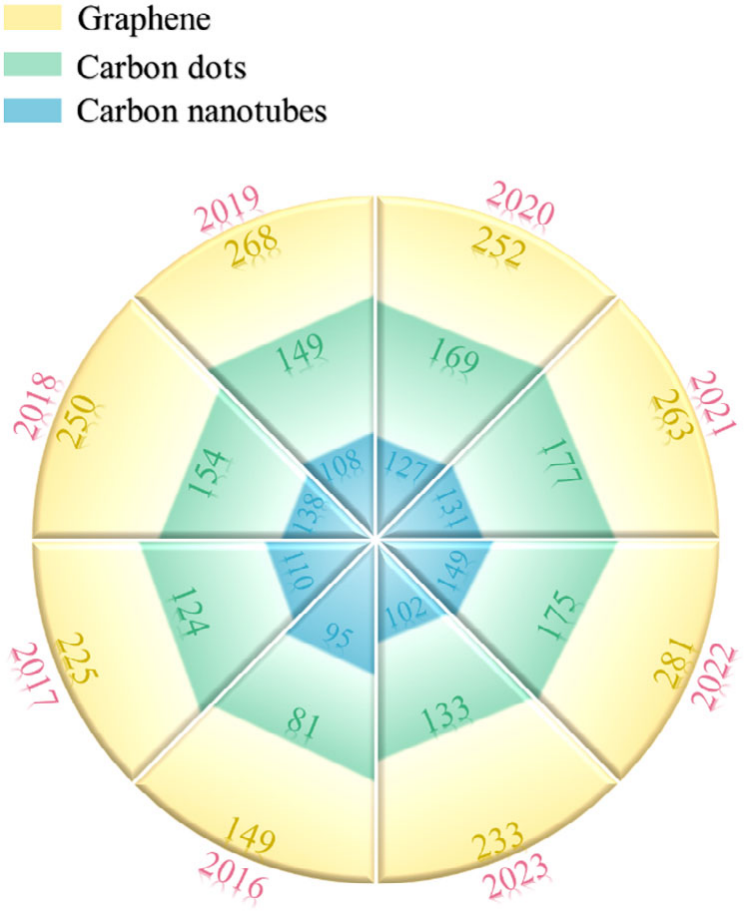
The number of journal publications on three carbon nanostructures in the last years. The data of publication numbers are taken from the Web of Science.

Even though several reviews have explored the potential of carbon‐nanomaterial‐based biosensors in medical diagnostics,^[^ ^85^, ^89^, ^96^, ^97^, ^98^, ^99^ ^]^ this field is highly active, and a large amount of research papers have been published recently. The number of publications on the three most predominant carbon nanostructures, i.e., graphene, carbon dots, and carbon nanotubes, depicts a booming trend in the last years, as shown in **Figure** **2**. Our review should be the first work to cover five different mechanisms of biosensors with the latest advancements in utilizing carbon‐based nanomaterials for cancer diagnosis applications and critically analyze the performance, advantages, and limitations of carbon nanomaterials across these mechanisms. A unique highlight of our work is the emphasis on the structure–property–performance correlation of carbon nanomaterials for suitable sensor architectures and high selectivity/sensitivity, guiding researchers toward the future design of high‐performing biosensors. To begin with, we elaborate on various prevailing biomarkers and cancer diseases, followed by introducing different sensing mechanisms and recent experimental realizations based on carbon‐based nanomaterials. The advantages of various carbon nanomaterials for different technologies have been emphasized.^[^ ^100^, ^101^ ^]^ At last, we conclude with an in‐depth discussion and a summary of the challenges associated with developing a practical technology for carbon‐nanomaterial‐based biosensors.

## 2 Types of Cancer Biomarkers

**Figure 3:**
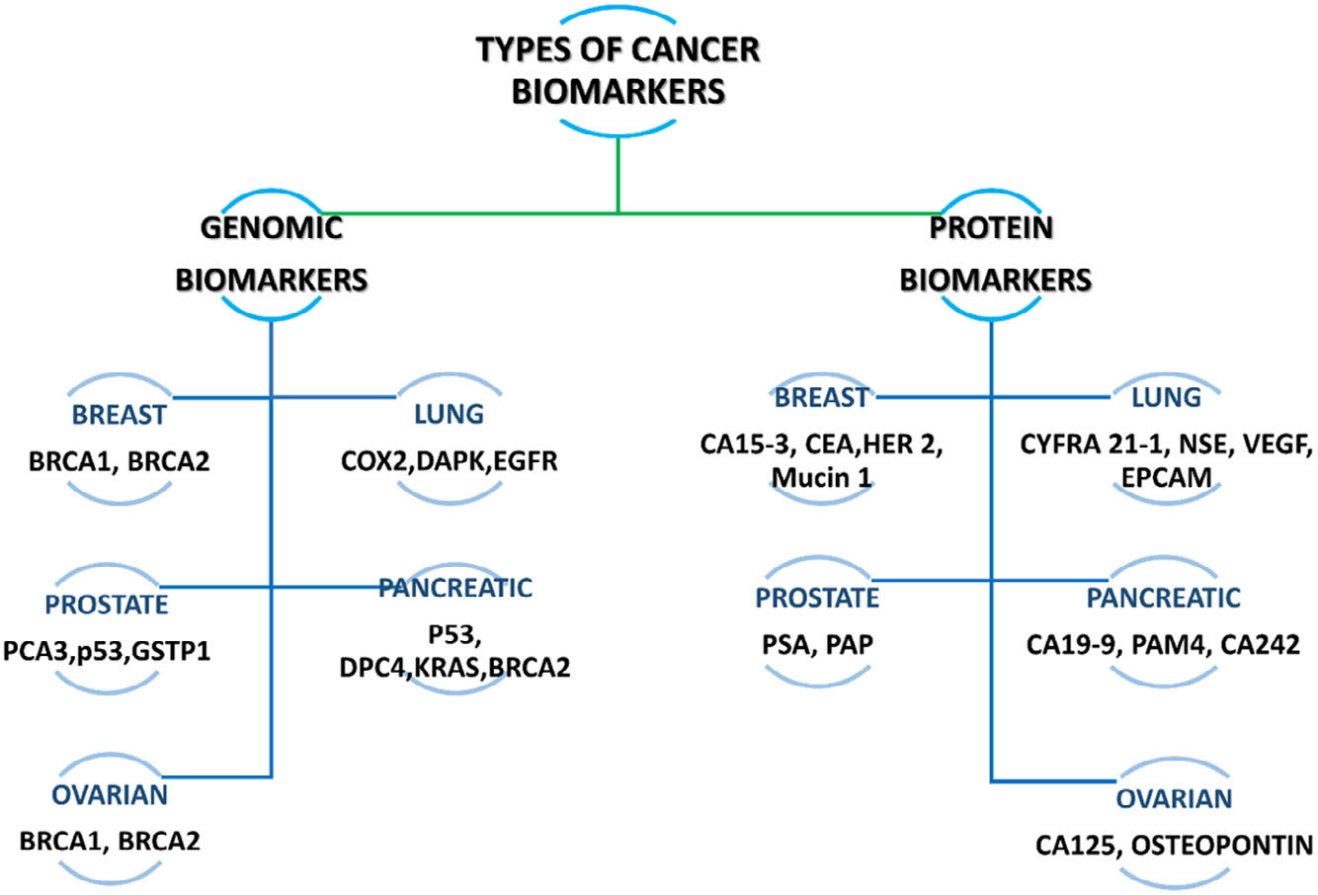
Classification of cancer biomarkers and typical examples. BRCA1, breast cancer gene 1; BRCA2, breast cancer gene 2; COX 2, cyclooxygenase 2; DAPK, death‐associated protein kinase; EGFR, epidermal growth factor receptor; PCA3, prostate cancer antigen 3; P53, tumor protein 53; GSTP1, glutathione S‐transferase pi 1; DPC4, pancreatic cancer deletion gene4; KRAS, Kirsten rat sarcoma virus; CA15‐3, carcinoma antigen 15‐3; CEA, carcinoembryonic antigen (CEA); HER 2, human epidermal growth factor receptor 2; Mucin 1, mucin short variant S1; CYFRA 21‐1, cytokeratin 19 fragment antigen 21‐1; NSE, neuron‐specific enolase; VEGF, vascular endothelial growth factor; EpCAM, epithelial cell adhesion molecule; PSA, prostate‐specific antigen; PAP, pulmonary alveolar proteinosis; CA19‐9, cancer antigen 19‐9; CA242, carbohydrate antigen 242; CA125, cancer antigen 125.

Biomarkers are biomolecules that have been widely used as identifiers for particular physiological processes or disease conditions.^[^ ^102^ ^]^ They can be found in biological fluids, including blood, serum, saliva, or tissues, making them essential tools for screening or diagnosing cancer.^[^ ^103^ ^]^ In the field of oncology, biomarkers have numerous potential applications, such as evaluating risk, conducting screenings, distinguishing between different diagnoses, determining prognosis, predicting treatment responses, and tracking disease progression.^[^ ^102^, ^103^ ^]^ Notably, with the advancement of analytical technology, a variety of new biomarkers have been developed and serve as diagnostic, predictive, and prognostic markers in cancer detection.^[^ ^103^ ^]^ Based on the molecular constituents of cancer biomarkers, this review will specifically focus on genomic biomarkers and protein biomarkers (**Figure** **3**).

### 2.1 Genomic Biomarkers

A genetic biomarker is an indicator for analyzing the changes in deoxyribonucleic acid (DNA) or ribonucleic acid (RNA) in normal biological and pathogenic processes.^[^ ^104^ ^]^ DNA biomarkers have been extensively employed to analyze DNA profiles and single nucleotide polymorphisms (SNPs) with abilities to identify the exact variations in genomic DNA.^[^ ^105^, ^106^ ^]^ The main advantage of DNA biomarkers is the ability to detect somatic mutation in patients efficiently. Somatic mutation or DNA sequence variations frequently occur in oncogenes, tumor suppressor genes, and mismatch repair genes.^[^ ^107^ ^]^ Therefore, DNA biomarkers, such as BRCA1 and BRCA2, can quickly determine these changes in DNA sequences related to disease states.^[^ ^108^ ^]^ On the other hand, RNA biomarkers are well known as transcriptomic biomarkers since they are able to detect the expression of RNA profiles covering a set of RNA molecules generated in the cells, such as mRNA, rRNA, tRNA, and other noncoding RNA (i.e., microRNA).^[^ ^109^, ^110^ ^]^ They represent a novel category of cancer‐specific biomarkers that are extensively used in fingerprint identification and clinical trials. The main advantages of RNA biomarkers lie in their solid ability to analyze the variation in posttranscriptional regulation of gene expression. Examples of RNA biomarkers include microRNAs such as miR‐21, miR‐122, miR‐16, and miR‐155.^[^ ^111^ ^]^

### 2.2 Protein Biomarkers

Protein biomarkers are of paramount significance due to the ubiquitous presence of proteins and their critical involvement in the metabolic and essential processes of both normal and tumor cells.^[^ ^112^ ^]^ Advanced mass‐spectrophotometry‐based proteomics has unveiled numerous potential protein biomarkers in the literature. Despite this, a small portion has obtained approval from the United States Food and Drug Administration (FDA).^[^ ^113^ ^]^ Compared to DNA and RNA biomarkers, protein biomarkers are often recognized with high sensitivity and specificity for low‐concentration detection.^[^ ^114^ ^]^ As a result, these biomarkers perform a vital role in disease recognition and dramatically slow the progression of the disease.^[^ ^115^ ^]^

In the early diagnosis of cancer, varieties of protein biomarkers have been exploited experimentally to detect disease states.^[^ ^116^ ^]^ For instance, alpha‐feto protein (AFP),^[^ ^117^ ^]^ carcinoembryogenic antigen (CEA),^[^ ^118^ ^]^ carbohydrate antigen 15 (CA 15‐3),^[^ ^119^ ^]^ prostate‐specific antigen (PSA),^[^ ^120^ ^]^ and New York esophageal squamous cell carcinoma‐1 (NY‐ESO1)^[^ ^121^ ^]^ are typical examples of protein biomarkers in cancer diseases.^[^ ^122^ ^]^ Among these, AFP is a glycoprotein with a size of 70 kDa, primarily produced in the fetal liver and yolk sac toward the end of the first trimester of pregnancy. AFP is found in the fetal serum with a concentration of 3000–5000 μg mL^−1^. A high level of AFP is found in newborns but deficient in healthy adults.^[^ ^123^ ^]^ After the age of 2 years, the expression of albumin‐like AFP is turned down with a drop in serum concentration of 5 × 10^−4^–1.5 × 10^−2^ μg mL^−1^.^[^ ^124^ ^]^ AFP is the most frequently utilized biomarker to detect hepatocellular carcinoma, the leading primary liver malignancy.^[^ ^125^ ^]^ AFP is used to detect multiple cancers, including pancreas, lung, colon, and ovaries or testicle cancer, during the diagnosis and treatment. It can also help determine the disease stage and observe the treatment response.^[^ ^126^ ^]^ The high level of AFP in serum is frequently linked to the presence of tumor.^[^ ^127^, ^128^ ^]^

CEA is a cell surface glycoprotein frequently employed in clinical trials. It is a 201 kDa glycoprotein secreted through the intestinal lumen, expressed at the apical portion of colonic epithelial cells, and released through the colonic lumen. CEA comprises a single polypeptide chain containing 641 amino acids, with lysine located at the N‐terminal position.^[^ ^129^, ^130^ ^]^ It is a glycoprotein usually produced by gastrointestinal tissue during fetal development.^[^ ^131^ ^]^ CEA can be expressed on the cell surface and circulate in the bloodstream.^[^ ^132^ ^]^ The average range of CEA in the blood is <2.5 ng mL^−1^. This protein produced by abnormal cells leads to cancer in a particular group.^[^ ^131^ ^]^ A high level of CEA could indicate certain types of cancers, like rectum, prostate, colorectal, ovary, lung, and liver cancer. Grunnet et al. illustrated that CEA was a tumor antigen first identified in tumor tissue extracts and found in fetal gastrointestinal tract epithelial cells.^[^ ^133^ ^]^

## 3 Most Common Types of Cancers and Their Biomarkers

Cancer is generally divided into five stages (0–IV).^[^ ^134^ ^]^ Stage zero (0) denotes cancer cells confined to the origin site and not extended to neighboring regions.^[^ ^135^ ^]^ This stage is usually treatable and is examined precancerous by health professionals. In stage I, cancer is limited to a small area without involvement of lymph nodes or adjacent tissues. Stage II is categorized as early‐stage cancer, indicating local growth but no spread. Stage III is characterized by significant growth and potential spread to lymph nodes or surrounding tissues. Notably, stage IV is referred as metastatic or advanced cancer, and it has already spread to different organs or regions of the human body.^[^ ^134^ ^]^ Different stages of cancer are also accompanied by the release or expression of specific biomarkers, cells, and metabolites in various body fluids, which can be used for early and rapid cancer diagnosis.^[^ ^136^ ^]^ As an effective detecting approach, biomarkers have significant potential in clinical use for estimating the risk of the disease and screening the primary stage of cancer or early diagnosis.^[^ ^137^ ^]^ As mentioned in Section 2.1 and 2.2, numerous genomic and protein biomarkers have been attempted for cancer diagnosis. In this section, we discuss in detail five common types of cancers, i.e., breast, ovarian, prostate, lung, and pancreas cancers and corresponding biomarkers that have been used to detect or confirm the presence of these diseases.

### 3.1 Breast Cancer

Breast cancer results from the abnormal proliferation of breast tissue cells. Typically, it originates from the epithelial lining cells of the ducts, lobules within the glandular tissue of the breast, or the surrounding connective tissues, and it has the potential to spread rapidly to the adjacent breast tissues.^[^ ^138^ ^]^ Invasive ductal carcinoma and invasive lobular carcinoma are the prevailing types of breast cancer. The former involves cancer cells spreading beyond the ducts into surrounding breast tissue, while the latter sees cancer cells spreading from lobules to other breast tissues. It is worth noting that in 2020, breast cancer affected over 2.3 million women, resulting in approximately 685 000 deaths worldwide.^[^ ^139^ ^]^ Thereby, effective diagnoses are warranted so patients can access treatment earlier and have improved prognoses.

BRCA1 and BRCA2 serve as major prognostic biomarkers in breast cancer detection.^[^ ^140^ ^]^ In many cases, the overexpression or mutation of BRCA1 and BRCA2 genes hints at the formation of breast cancer. These genetic biomarkers are mainly applied to predict high‐risk hereditary breast cancer.^[^ ^141^ ^]^ More importantly, genetically unstable BRCA genes in which physical deletion of wild‐type alleles can result in breast cancer.^[^ ^142^ ^]^ BRCA1 is a tumor suppressor gene or anti‐ancogene that contains a protein of 1863 amino acids, which control transcriptional activation, DNA repair, apoptosis, and chromosomal remodeling. Similarly, the BRCA2 produces a protein of 3418 amino acids that helps to repair damaged DNA and modulate transcriptional activation and cell growth.^[^ ^143^ ^]^ The inactivation or physical deletion of BRCA2 leads to the tumor by duplication of the mutant allele, gene conversion, and mitotic recombination.^[^ ^142^ ^]^

Biomarkers linked to breast cancer at the protein level encompass CA, CA 15‐3, CA 27‐29, CEA, estrogen receptor (ER), progesterone receptor (PR), and HER2.^[^ ^30^ ^]^ Among them, CA15‐3 and HER2 are standard biomarkers for breast cancer patients. CA15‐3 is the most often utilized biomarker for patients with symptoms.^[^ ^144^ ^]^ It is a carbohydrate‐containing protein biomarker called Mucin, a large transmembrane glycoprotein, highly *O*‐linked glycosylated protein with extracellular domains consisting of highly conserved 20 repeated units of amino acids.^[^ ^145^ ^]^ CA 27‐29 is a breast cancer‐associated antigen produced by the MUC 1 gene. It is worth mentioning that up to 80% of women's cancer is due to overexpression of CA 27‐29 protein.^[^ ^146^ ^]^ HER2 is usually expressed in organs’ epithelial cells, and it is also known as Neu, featuring an extracellular binding domain, a single transmembrane domain (E), and an intracellular tyrosine kinase region.^[^ ^147^ ^]^ The receptor of HER2 takes part in the proliferation of cells and cell‐to‐cell communication through signal transduction that affects the transcription of genes by the phosphorylation process.^[^ ^148^ ^]^ Notably, HER2 can be found in breast tumor cells at a low level, in which HER2 receptor protein increases the cell growth and malignant growth of breast cancer cells overexpressed in ≈20–30% of breast cancers, enabling it to breast cancer detection in the earliest stages.^[^ ^149^ ^]^ Therefore, HER2 protein is considered a significant predictive biomarker for monitoring the presence and aggressiveness of breast cancer cells.

### 3.2 Ovarian Cancer

Ovarian cancer typically originates in one of the three types of ovarian tissue, namely epithelial, stromal, or germ cell tissue, with epithelial tumors accounting for 90% of all ovarian cancer cases.^[^ ^150^ ^]^ Ovarian cancer protein biomarkers comprise CA‐125, osteopontin, CEA, vascular endothelial growth factor (VEGF), and human epididymis protein 4 (HE4). Moreover, genetic markers involve germline variations in both BRCA1 and BRCA2 genes, which may substantially increase ovarian cancer's lifespan threat.^[^ ^151^ ^]^

BRCA 1 and BRCA 2 biomarkers are also tumor suppressor genes in ovarian cancer as a mutation of these genes may increase the risk of ovarian cancer progression.^[^ ^152^ ^]^ CA‐125 is overexpressed and presents abnormally in ovarian cancer patients.^[^ ^152^ ^]^ To date, the CA‐125 biomarker blood test has become a routine tool for ovarian monitoring and high‐risk group detection, in which a high level of CA‐125 indicates a high possibility of ovarian cancer.^[^ ^153^ ^]^ Nowadays, researchers have already achieved a low limit of detection (LOD) of CA‐125 smaller than 26 U mL^−1^.^[^ ^154^ ^]^ Nevertheless, the exact detection rate is still unclear because the majority of the patients have a low value at early detection. In contrast, HER4 is a standard biomarker providing high sensitivity and specificity for diagnosing and progressing ovarian cancer with a detection limit of 55 pmol L^−1^.^[^ ^155^ ^]^ Additionally, HER4 protein was known to induce tumor progression in both breast and ovarian cells,^[^ ^156^, ^157^ ^]^ and its serum biomarker was used to diagnose early‐stage ovarian cancer.^[^ ^158^ ^]^ Osteopontin is a glycophosphoprotein produced by T lymphocytes, macrophages, and leucocytes, and it is commonly found in the extracellular matrix, inflammation sites, and body fluids in ovarian cancer.^[^ ^159^, ^160^ ^]^ VEGF stimulates endothelial cell proliferation and acts as a mediator of angiogenesis in this cancer, with a relatively low LOD of 0.5 pg mL^−1^.^[^ ^161^, ^162^ ^]^

### 3.3 Pancreatic Cancer

Pancreatic cancers primarily occur in two main types: exocrine pancreatic cancer and neuroendocrine pancreatic cancer.^[^ ^163^, ^164^ ^]^ Adenocarcinoma, commonly known as ductal carcinoma, is an exocrine cancer that predominantly arises in the lining of the pancreatic ducts. On the other hand, pancreatic neuroendocrine tumors (NETs) originate from cells in the pancreatic endocrine gland.^[^ ^165^ ^]^ In this regard, the cancer antigen 19‐9 (CA19‐9) test becomes the standard diagnostic blood test for pancreatic cancers. The high expression level of CA19‐9 in the blood is an indicator for patients with pancreatic cancer symptoms.^[^ ^166^ ^]^ CA19‐9 and pulse amplitude modulation 4‐level (PAM4) are specific protein‐based biomarkers for pancreatic cancer. Some other serum protein biomarkers are linked to the pancreatic disease diagnosis, including MUC1, MUC4, CEA, CAM1, MMP‐9 (OPG), osteopontin (OPN), CA 242, CA 50, CEA, CA 72‐4, and CA 494.^[^ ^167^ ^]^ Among these biomarkers, CA19‐9 has been approved for pancreatic cancer detection by the FDA.^[^ ^168^ ^]^ According to Thapa et al., the lowest detection limit of CA19‐9 is around 37 U mL^−1^, providing sensitivity of 70–90% and selectivity of 68–91% in the serum sample.^[^ ^169^ ^]^ Similarly, CA 242 is an indicator for pancreatic cancer diagnosis and gives a sensitivity of 74% and selectivity of 9 L%, which is slightly lower than CA19‐9.^[^ ^170^ ^]^ P53, deleted in pancreatic carcinoma 4 (DPC4), p16, and BRCA2 are a few common signature genetic biomarkers for pancreatic cancer, in which they detect the mutation of particular genes such as chromosomal instability and telomere abnormalities in most patients.^[^ ^171^ ^]^

### 3.4 Prostate Cancer

In Australia, prostate cancer stands as the third most common cause of death attributed to cancer and remains the second most prevalent disease among men.^[^ ^19^, ^20^, ^22^, ^172^ ^]^ It is classified as either acinar adenocarcinoma or ductal adenocarcinoma. Acinar adenocarcinoma originates from gland cells that line the prostate gland, whereas ductal adenocarcinoma tends to spread at a faster rate than acinar adenocarcinoma, which arises in cells lining the ducts of the prostate gland.^[^ ^173^ ^]^ PSA and prostatic acid phosphatase (PAP) are protein‐based biomarkers specifically associated with prostate cancer. PSA is usually present in body fluids, and a high PSA level can hint at prostate cancer.^[^ ^174^ ^]^ Therefore, PSA is utilized as the primary tumor biomarker for the early diagnosis of prostate cancer in clinical settings. Typically, a blood PSA level higher than 4.0 ng mL^−1^ is considered high risk.^[^ ^175^ ^]^ On the other hand, genetic markers of prostate cancer include prostate cancer antigen 3 (PCA3), p53, and glutathione‐S‐transferase P1 (GSTP1).^[^ ^176^ ^]^ Abnormal cancer cells circulating in the bloodstream are used as a detection modality, which allows for a shorter detection period for tumor cells in the blood.^[^ ^177^ ^]^ E‐cadherin is a serum‐based biomarker for prostate cancer, and it acts as a mediator of cell‐to‐cell adhesion or cell–cell signalling.^[^ ^178^ ^]^ Leman and Getzenberg reported that numerous genes were involved in cell adhesion and controlled prostate cancer cell growth, in which E‐ cadherin showed relatively low activity in prostate cancer than in normal tissue.^[^ ^178^ ^]^ Typically, the mutation of tumor suppressor genes such as p53 and phosphatase with TENsin homology (PTEN), which regulate cell growth, can contribute to the advancement of prostate cancer.

### 3.5 Lung Cancer

Lung cancer is one of the most prevalent forms of tumors and continues to be the primary contributor to cancer‐related deaths, responsible for 18.4% of all total fatalities.^[^ ^21^, ^179^ ^]^ Lung cancer can be classified into two main types: small cell lung cancer (SCLC) and non‐small cell lung cancer (NSCLC). NSCLC is the more common type, accounting for over 80% of cases. It is further divided into three subtypes: large cell carcinoma, adenocarcinoma, and squamous cell carcinoma. Conversely, SCLC comprises about 20% of cases and can be categorized into subtypes, including small cell carcinoma, mixed small cell/large cell carcinoma, and combined small cell carcinoma.^[^ ^180^ ^]^ The leading cause of lung cancer is smoking tobacco and drinking alcohol, although environmental pollutants and genetic factors also play a role in the incidence of lung cancer.^[^ ^181^ ^]^

Several noninvasive approaches have been devised for diagnosing lung cancer, including ultrasound, MRI, PET imaging, low‐dose helical CT scans, and bone studies. Invasive approaches such as sputum cytology, bronchoscopy, and needle biopsy are also employed for diagnosis.^[^ ^180^ ^]^ These conventional methods, however, cannot detect lung cancer at an early stage because their detecting principles rely on the tumor's phenotypic properties. Biomarkers have emerged as promising tools for early‐stage detection of lung cancer, and a diverse range of lung cancer biomarkers have been documented in scientific literature. Genomic‐based biomarkers encompass various types, such as cyclooxygenase‐2 (COX‐2), interleukin‐8 (IL‐8) mRNA, death‐associated protein kinase (DAPK), polypyridyl complexes 3 (PRCS3), p53 mutant, and epidermal growth factor receptor (EGFR), with particular emphasis on c‐ErbB‐1 and c‐ErbB‐2.^[^ ^182^ ^]^ Neuron‐specific enolase (NSE), progastrin‐releasing peptide (ProGRP), cytokeratins 21‐1 (CYFRA 21‐1), epithelial cell adhesion molecule (EPCAM), CEA, and VEGF are essential protein biomarker for lung cancer diagnosis.^[^ ^183^, ^184^ ^]^ Okamura et al. demonstrated that the sensitivity and specificity of the CEA biomarker could be up to 69% and 68%, respectively, while these performance values of CYFRA 21‐1 were 43% and 89%, respectively.^[^ ^185^ ^]^ The minimum detectable levels for NSE, ProGRP, CYFRA 21‐1, and VEGF were 0.05, 63, 0.32 ng mL^−1^, and 31.25 pg mL^−1^, respectively. Additionally, CA 125 and CA 19‐9 are also utilized in lung cancer diagnosis.^[^ ^182^ ^]^

Lung cancer treatment is known for its unique challenges due to factors such as early detection, stage at diagnosis, and treatment options. Recently, the synergistic combination of biosensors and biological markers has played an important role in lung cancer diagnosis and treatment.^[^ ^186^ ^]^ Zamay et al. demonstrated that CEA, CYFRA 21‐1, and ProGRP could serve as clinical biomarkers for lung cancer diagnosis, while they could not be functional alone for lung cancer detection due to their low concentration level in serum.^[^ ^187^ ^]^ To this end, the combination of two biomarkers (e.g., CEA and CYFRA 21‐1) could significantly enhance the sensitivity and specificity for disease detection.^[^ ^188^ ^]^

## 4 Multifunctional Carbon‐Based Nanomaterials for Biosensing

**Figure 4:**
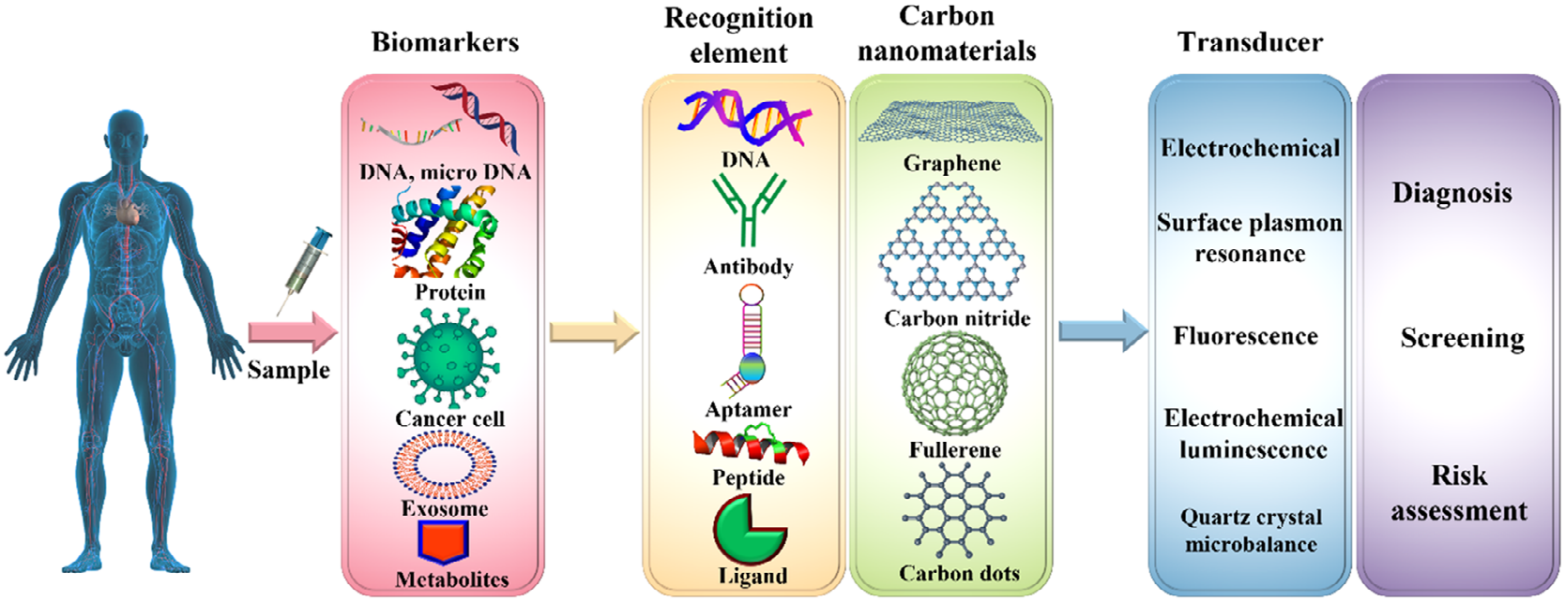
Schematics of the working process of carbon‐nanomaterial‐based biosensors for early cancer diagnosis.

Biosensors are a type of high sensitive device capable of detecting various biological analytes.^[^ ^189^ ^]^ According to the International Union of Pure and Applied Chemistry (IUPAC), a biosensor is a self‐contained integrated system with a bioreceptor‐transducer system, capable of delivering quantitative analytical information through bioreceptors like enzymes, DNA probes, and antibodies.^[^ ^190^, ^191^ ^]^ The bioreceptor is a biomolecule that recognizes the target analyte, and the transducer can convert the recognized information into quantitative signals, as demonstrated in **Figure** **4**.^[^ ^192^ ^]^ However, the conventional methods of detecting biomarkers, like immunological assays and biopsies, suffer from low sensitivity, long detection time, and are hazardous to health with the necessity of sophisticated instruments and trained operators. Therefore, the development of highly effective biosensors for cancer diagnosis is a major challenge.

Recently, nanomaterials, especially carbon‐based nanostructures, have been highly effective for sensing cancer biomarkers because of their increased specific surface area, surface binding sites, and electrochemical properties.^[^ ^193^ ^]^ The surface active sites of these nanostructures can be used for selectively binding/sensing different biomolecules, including antigens, peptides, and other cancer‐specific biomarkers. In this section, we will discuss various carbon‐based biosensors, including ECL, electrochemical, fluorescence, QCM, and SPS biosensors.

### 4.1 Electrochemiluminescence Biosensors

ECL, also known as electrogenerated chemiluminescence, is a highly efficient analytical technique employed in various fields.^[^ ^194^ ^]^ ECL is based on the generation of electro‐active species near the electrode surface, followed by high‐energy electron‐transfer reactions that lead to the formation of excited states, resulting in light emission.^[^ ^195^ ^]^ As the light is produced from the biomolecules (luminophores), the concentration of biomolecules can be detected from the intensity of emission light.^[^ ^196^, ^197^ ^]^ Luminophores can be the atom or functional group in a chemical compound responsible for having luminescent or light‐emitting properties and are typically loaded on biomolecules like antigens and antibodies.^[^ ^198^ ^]^ Semiconductor QDs, Ru(bpy)_3_^2+^, luminol, and their analogs are frequently employed as ECL luminophores. Compared to other sensing methods, ECL is a sensitive and potential method that can efficiently analyze cancer biomarkers in clinical samples.^[^ ^199^, ^200^ ^]^ The high sensitivity, low background, and ease of control are the signature characteristics of this type of biosensor.^[^ ^201^ ^]^ Over the past decade, ECL techniques utilizing carbon nanomaterials have been extensively investigated owing to their numerous advantages. These nanomaterials demonstrate remarkable conductivity of electricity, consistent electrochemical properties, and a comparatively high specific surface area, contributing to their high ECL activity. Additionally, the availability of surface functional categories and defects in their low‐dimensional structures enables versatile functionalization, making them a novel class of luminescent electrode materials suitable for quantitative analysis of biomolecules such as DNAs, enzymes, and cancer proteins.^[^ ^202^, ^203^ ^]^

#### 4.1.1 Graphene‐Based ECL Biosensors

As the most famous carbon nanomaterial, graphene has been most investigated for ECL‐based cancer biosensors. The contribution of graphene boosts the performance of the ECL sensing system originating from its extraordinary physical properties.^[^ ^204^, ^205^ ^]^ In many cases, graphene may increase the surface area of sensing platforms, improve chemical processes (such as the binding of biomolecules), speed up the passage of electrons across electrode interfaces, and amplify electron signals during sensing.^[^ ^206^ ^]^ The efficient binding of antigen–antibody takes place on the graphene surface layer and exhibits high‐accuracy detection of particular analytes.^[^ ^207^ ^]^ These properties enable graphene for high‐accuracy sensing in ECL immunosensors and as carriers to load ECL labels on biomolecules. For example, Wu and co‐workers constructed SnO_2_/reduced graphene oxide (rGO)/gold nanoparticles (AuNPs) composites for determining the presence of insulin, using the SiO_2_@Polydopamine as the ECL signal quencher.^[^ ^208^ ^]^ In this system, SnO_2_/rGO behaved as the support material and helped to load a high density of AuNPs owing to its high stability, good reversible capacity, and high specific surface area, while AuNPs were responsible for combining with antibodies by forming Au–N covalent bond.

Graphene can also act as an amplifier that improves the rate of electron transfer at the electrode. In this case, the graphene‐modified electrode in the ECL biosensing system promotes a higher sensitivity of cancer biomarkers than other detection techniques.^[^ ^209^ ^]^ For instance, Zhang et al. described an ECL biosensor utilizing graphene functionalized with a gold–silver nanocomposite, in which graphene substantially increases the surface area, enabling greater binding of primary antibodies and accelerating electron transfer rates.^[^ ^210^ ^]^ Furthermore, the secondary antibodies labeled with CdTe QDs‐coated carbon microspheres amplified ECL intensity and stabilized luminescence properties, enabling accurate and highly sensitive CA 125 detection with satisfied linear relationships ranging from 0.008 to 50 U mL^−1^. Similarly, Wang et al. introduced an ECL sensor with CdSe QDs/GO‐chitosan for cytochrome C detection.^[^ ^211^ ^]^ In this system, the high ECL sensitivity stems from the synergistic effects of three components: GO guarantees high stability and conductibility, and its porous structure not only enables a great rough particular surface area to load significant quantities of CdSe QDs but also promotes the diffusion of K_2_S_2_O_8_ into the interface of electrode and accelerate reaction; chitosan (CS) with amino and hydroxy groups enhances the stability of the composites because of its excellent water permeability and good biocompatibility; CdSe QDs reacts with K_2_S_2_O_8_ and generates ECL emission. The fabricated ECL sensor exhibited a high sensitivity for cytochrome C spanning from 4.0 to 324 μM, coupled with LOD 1.5 μM.

**Figure 5:**
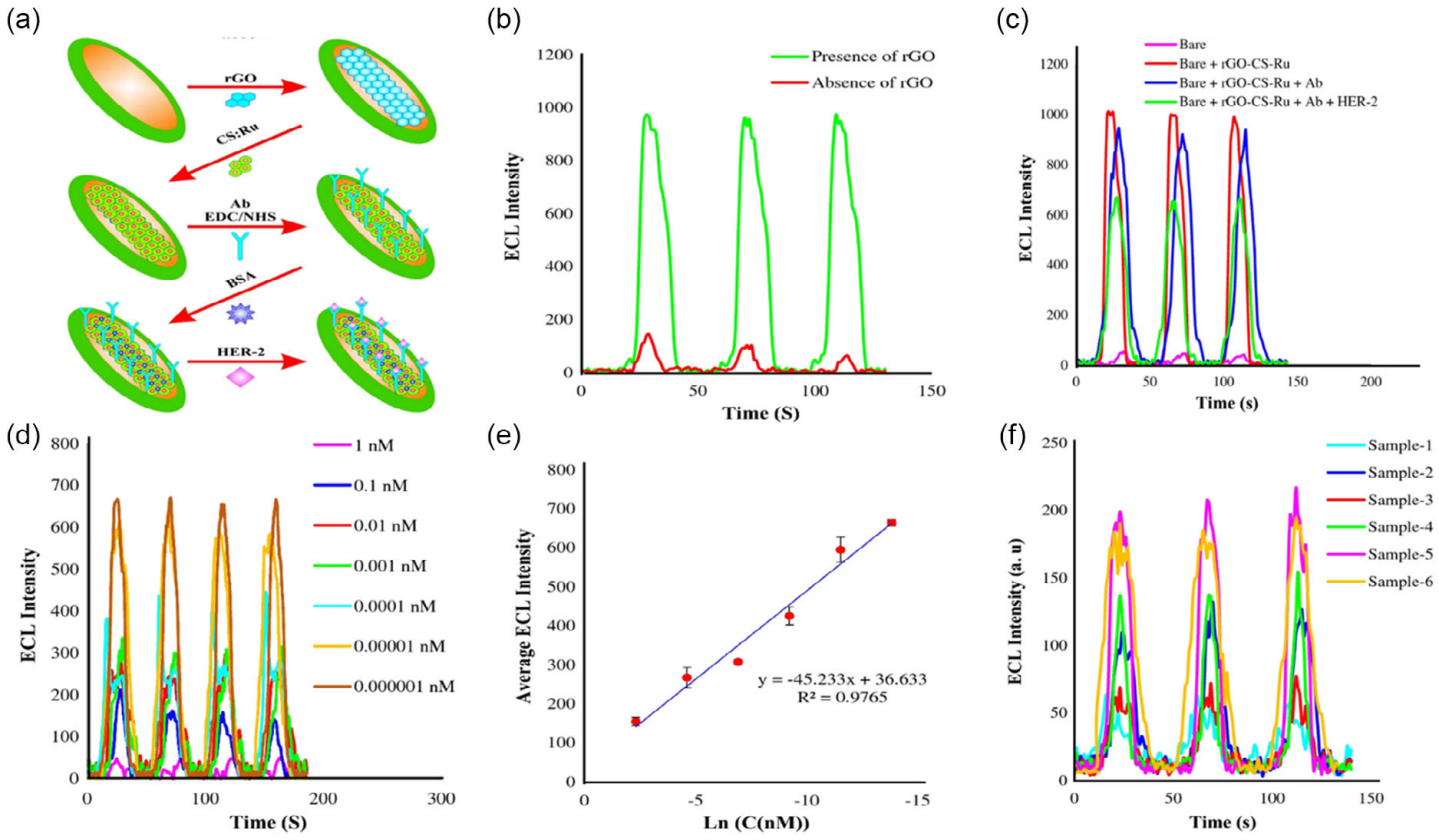
a) Graphical representation of rGO‐based immunosensor for HER‐2 protein. b) Effect of rGO on the intensity of ECL signals. c) The three consecutive ECL graphs of Bare, Bare + rGO‐CS‐Ru, Bare + rGO‐CS‐Ru+Ab, and Bare + rGO‐CS‐Ru + Ab+HER‐2. d) The ECL curves for GCE/rGO/CS‐[Ru(bpy)_3_]^2+^/Ab‐HER‐2 for different concentrations of HER‐2 protein (0.000001 to 1 nM). e) Calibration curve of ECL intensity of the final modified electrode at the corresponding concentrations of HER‐2 protein. f) ECL plots of investigation of the HER‐2 in untreated serum samples from breast cancer patients. a–f) Reproduced under the terms of the CC‐BY Creative Commons Attribution 4.0 International license (https://creativecommons.org/licenses/by/4.0).^[^ ^214^ ^]^ Copyright 2021, The Authors, published by Springer Nature.

The ECL‐based biosensor, employing a graphene/Au‐CdS:Eu QDs architecture, effectively detected AFP biomarkers as well.^[^ ^212^ ^]^ Indeed, the ECL immunosensor adopts a dual‐quenching approach, with horseradish peroxidase molecules on Au/Ab2 initiating the redox interaction involving substrate hydroquinone and H_2_O_2_, resulting in ECL intensity quenching as the concentration of co‐reactant H_2_O_2_ decreases. Additionally, the ECL resonance energy transfer (ECL‐RET) phenomenon takes place between the Au and CdS:Eu QDs, resulting in additional ECL intensity quenching owing to the spectral overlap between the two components. Specifically, the emitted light from rGO/Au/CdS:Eu QDs (donor) is reabsorbed by Au nanorods (acceptor).^[^ ^213^ ^]^ Consequently, this ternary system exhibited the linear range of AFP detection between 0.05 pg mL^−1^ and 1 ng mL^−1^ at LOD of 0.05 pg mL^−1^. It also demonstrated a 96% to 110% restoration of AFP in serum. Similarly, Nasrollahpour et al. conducted a label‐free ECL immunosensor assay on a rGO and CS composite to detect the presence of the HER‐2 protein in breast cancer.^[^ ^214^ ^]^ Initially, rGO was electrodeposited on the surface of a glassy carbon electrode (GCE) using cyclic voltammetry (CV). Subsequently, a biopolymer solution of CS/[Ru(bpy)_3_]^2+^ was coated on the modified electrode, with CS covalently interacting with rGO. Monoclonal antibodies (Abs) specific to HER‐2 were attached to the amine groups of CS/[Ru(bpy)_3_]^2+^/rGO/GCE through amide bonds using EDC/NHS chemistry (**Figure** **5**a). The efficiency of the rGO electrode was evaluated, and ECL signals were recorded with/without rGO electrodeposition (Figure 5b). The presence of rGO significantly enhanced ECL intensities, while the absence of rGO led to very low ECL intensities, indicating substantial quenching of emitted materials. The various steps in the electrode separation process of the ECL sensor are depicted in Figure 5c. The ECL intensity decreased when the Abs and the target HER‐2 protein (1 fM) were introduced onto the electrode, which can be attributed to steric hindrance that reduced the ECL intensity due to the tripropylamine (TPrA) obstruction from reaching the electrode surface. The ECL calibration curve, obtained using GCE/rGO/CS/[Ru(bpy)_3_]^2+^, included seven different concentrations of HER‐2 protein (ranging from 1 fM to 0.000001 nM) (Figure 5d **)**. The sensitivity of the detection is determined by the slope of the calibration curve (*y* = −45.233*X* + 36.633) (Figure 5e). This result demonstrated that the developed technique exhibited high sensitivity in detecting HER‐2 protein. The prepared immunosensor was validated by analyzing several real samples collected from breast cancer patients at different stages. To assess the reliability of the system, the obtained results were compared with pathological examinations, as shown in Figure 5f.^[^ ^214^ ^]^

#### 4.1.2 g‐C_3_N_4_‐Based ECL Biosensors

Apart from graphene, g‐C_3_N_4_ with highly flexible electronic structures was also intensively investigated for ECL biosensor applications.^[^ ^215^, ^216^, ^217^, ^218^ ^]^ In 2014, a pioneered work by Li et al. first employed g‐C_3_N_4_ as a luminophore to construct a label‐free g‐C_3_N_4_/graphene‐based ECL biosensor for squamous cell carcinoma antigen (SCCA) detection.^[^ ^219^ ^]^ In this study, g‐C_3_N_4_ was closely anchored on the surface of graphene through electrostatic interactions, where graphene was utilized to increase the sensitivity of the ECL sensor further. The introduction of SCCA and its corresponding antibodies resulted in a linear reduction of ECL intensity, encompassing the span from 0.025 to 10 ng mL^−1^, and featuring an LOD of 8.53 pg mL^−1^.

**Figure 6:**
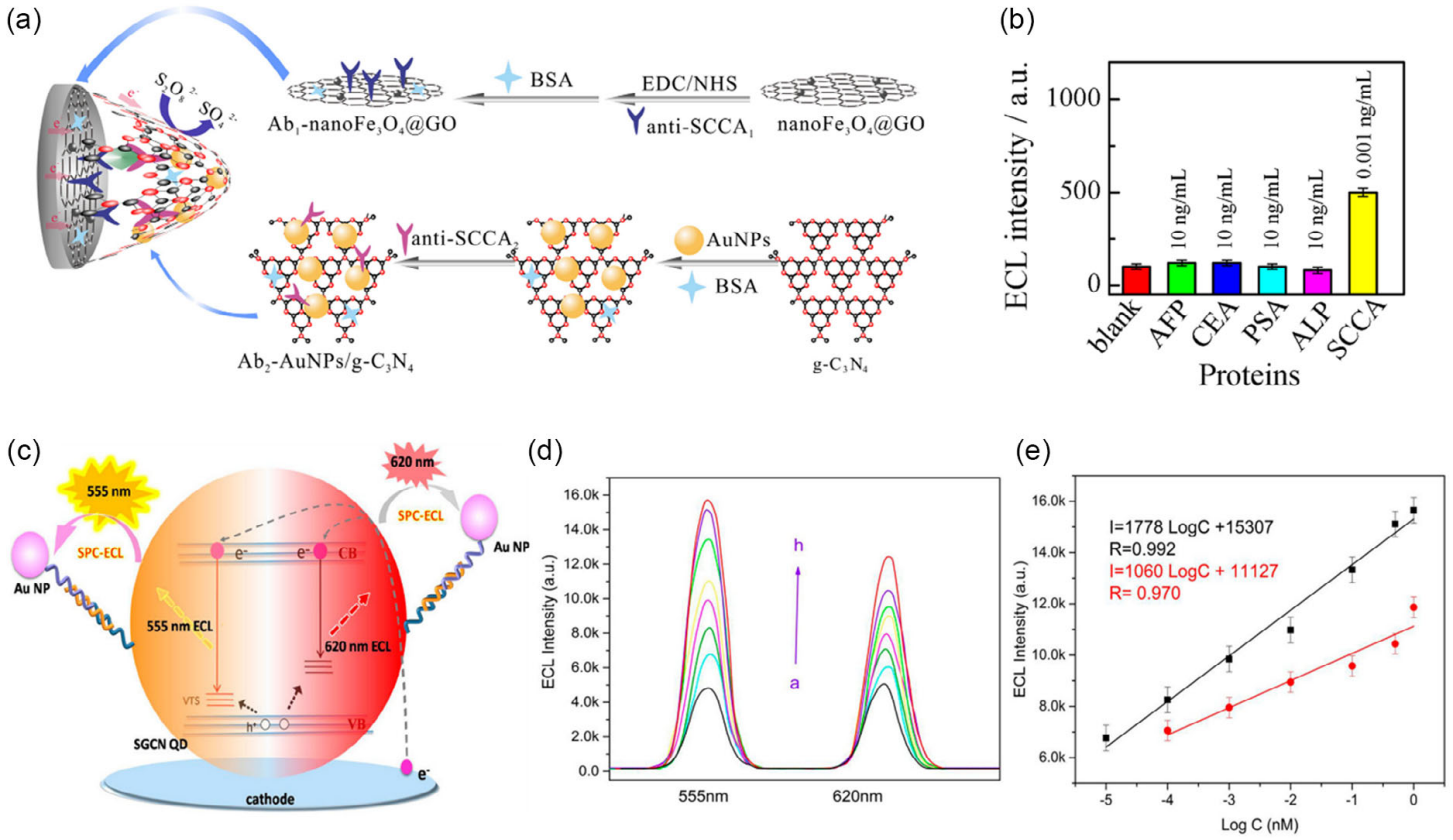
a) Schematics of fabrication procedure of the Ab_1_‐Fe_3_O_4_@GO and Ab_2_‐AuNPs/g‐C_3_N_4_ bio‐conjugates. b) The selectivity of the ECL biosensor in the blank, AFP, CEA, PSA, ALP, and SCCA. a,b) Reproduced with permission.^[^ ^220^ ^]^ Copyright 2016, Elsevier. c) Schematic representation of S‐g‐CNQDs dual‐band ECL biosensor mechanism. d) Relationship between the ECL intensity and different concentrations (0, 50 fM, 0.1, 1 pM, 0.01, 0.1, 0.5–1 nM at 555 and 620 nm) of K‐RAS gene. e) The corresponding linear relationship of the ECL intensity and K‐RAS gene concentrations. c–f) Reproduced with permission.^[^ ^229^ ^]^ Copyright 2019, American Chemical Society.

Shortly after this work, Wu et al. developed an innovative ECL biosensor of the “in‐electrode” type to detect SCCA, employing AuNPs/g‐C_3_N_4_ and nanoFe_3_O_4_@GO.^[^ ^220^ ^]^ In this system, the nanoFe_3_O_4_@GO and the SCCA primary antibodies (Ab_1_) played the role of capture probe, and AuNPs/g‐C_3_N_4_ labeled the Ab_2_ sary antibodies behaved as the signal tag (**Figure** **6**a). In this case, AuNPs enhanced the ECL intensity of g‐C_3_N_4_ thanks to the increased conductivity and accelerated electron transfer rate between the electrode and g‐C_3_N_4_. After Fe_3_O_4_@GO catches SCCA at the Ab_1_ site, the Ab_2_‐AuNPs/g‐C_3_N_4_ in the immunocomplex can be conjugated sequentially with the antigen and thereby fixed on the surface of the electrode. The immunological reaction between SCCA and its antibody demonstrated high sensitivity and selectivity, yielding a low LOD of 0.4 pg mL^−1^, as shown in Figure 6b. Additionally, the same research group designed an ECL immunosensor based on g‐C_3_N_4_ for the detection of CA125, a marker linked to ovarian cancer. Through the application of amino‐coated Fe_3_O_4_ nanoparticles and anti‐CA125 onto single‐use screen‐printed carbon electrodes, they enabled efficient electron transfer between g‐C_3_N_4_ and the electrode, resulting in a notable augmentation of ECL intensity.^[^ ^221^ ^]^

Fruitful achievements have been reported in g‐C_3_N_4_‐based nanocomposites ECL systems, including promising LOD values.^[^ ^222^ ^]^ For example, the CA125 could be detected by Anti‐CA125‐nanoFe_3_O_4_@g‐C_3_N_4_ composite at concentrations between 0.001 and 5 U mL^−1^, with an LOD of 0.4 mU mL^−1^.^[^ ^223^ ^]^ Idris et al. discovered that g‐C_3_N_4_ nanosheets/gold electrodes could form an ultrasensitive ECL immunosensor for the detection of DNA with LOD of 0.001 fg mL^−1^.^[^ ^224^ ^]^ Similarly, Liu et al. designed an ECL sensor utilizing graphitic QDs (g‐CNQDs)/AuNPs, demonstrating an impressive LOD of 0.01 fM for DNA.^[^ ^225^ ^]^ Undoubtedly, g‐C_3_N_4_ is a potential ECL luminophore and can open an avenue for future biosensor applications.

#### 4.1.3 Carbon‐Dot‐Based ECL Biosensors

Carbon dots (CDs) are another effective ECL luminophores widely used in detection technologies because of the carboxylic acid groups on their surface. The inherent fluorescence of CDs can be significantly suppressed due to significant electron transfer transpiring at the interface between biomolecules and CDs. This characteristic renders CDs highly promising for biomolecule detection, such as nucleic acids, amino acids, and vitamins, as well as various biomarkers like cancer cells, cancer antigens, and microRNA.^[^ ^226^ ^]^ Remarkably, they can play the roles of both luminophores and quenchers. In most cases, CDs serve as novel luminescent reagents owing to their ECL properties. For example, Chai's team fabricated a novel ECL biosensor using amino‐modified CDs and TiO_2_NPs functionalized with AuNPs (CDs‐Au‐PEI‐TiO_2_) for miRNA‐21 detection.^[^ ^227^ ^]^ Original CDs‐Au‐PEI@TiO_2_ composites presented a strong initial ECL signal due to the effective immobilization of CDs by PEI@TiO_2_ as support, while the target miRNA‐21 presence repeatedly caused hybridization of H1, which significantly reduced the ECL intensity with the miRNA‐21 concentrations between 0.1 fM and 10 pM, with an LOD of 0.03 fM. In a separate investigation, Sun and his team integrated graphene QDs with molybdenum disulfide (MoS2‐GQDs) to design an ECL biosensor with an “on/off” switch, enabling the detection of specific DNA sequences.^[^ ^228^ ^]^ Interestingly, the combination of 2D MoS2 nanosheets and long‐chain hairpin DNA significantly increased the loading capacity of GQDs. During the ECL process, charge transfer occurred between GQDs and MoS_2_, resulting in the “on” phase. Simultaneously, exonuclease III and target DNA were more easily bound by DNA walker cyclic amplification, leading to increased ECL intensity. On the other hand, DNA2‐Fc‐DNA1 served as a quencher for the ECL signal, resulting in an “off” phase. The newly developed ECL biosensor had an outstanding linear detection range of 1 nM to 100 aM and a remarkable LOD of 25.1 aM. In 2019, Zhang et al. constructed a wavelength‐dependent dual‐band biosensor by using sulfur‐doped graphitic phase g‐C_3_N_4_ QDs (S‐g‐CNQDs) to identify the K‐RAS gene, a genomic biomarker linked to pancreatic cancer, using surface plasmon coupling ECL (SPC‐ECL) detection.^[^ ^229^ ^]^ As depicted in Figure 6c, the ECL signal at 620 nm is similar to common g‐CNQDs or other semiconductor materials, which is related to its conduction band and valence band. However, another ECL signal at 555 nm should be ascribed to the sulfur doping vacancies, which generate an adding energy trap state positioned above the valence band of QDs, effectively accepting holes. Since an improved spectrum overlapping of 555 nm peak with the surface plasmon absorption peak of AuNPs, the ECL intensity at this wavelength shows a much higher enhancement (Figure 6d), demonstrating a wider linear range between 50 fM and 1 nM, with an LOD of 16 fM at 555 nm, compared to these of 0.1 pM to 1 nM and 30 fM at 620 nm (Figure 6e).

Notably, CDs can also act as quenching materials in ECL systems. For instance, in 2020, Yang's team first employed CDs to dampen the ECL signal of a Ru(bpy)_3_^2+^‐tripropylamine system.^[^ ^230^ ^]^ In this case, CDs connected with complementary DNA were attached to the electrode surface and acted as a quenching probe. Without the target miRNA 142‐3p, CDs‐DNA stayed at the electrode and caused weak ECL intensity; with miRNA 142‐3p, on the other hand, DNA could bind with targets and form DNA/RNA duplexes. More importantly, DNA would be hydrolyzed afterward, and the target RNA could be released again and bind with other DNAs. This repeated hydrolyzed DNA process can effectively remove CDs‐DNA composites from the electrode surface, thus resulting in a much‐enhanced ECL intensity and a sensitive platform for detecting microRNA 142‐3p with a linear range between 10 fM and 1.0 nM, with an LOD of 10 fM. **Table** **1** summarizes various reports on ECL biosensors based on carbon materials for cancer detection.

**Table 1:** Summary of ECL biosensors used to detect cancer biomarkers

Carbon materialsa)	Cancer types	Biomarkers	LOD	Refs.
Au/Ag‐rGO/aminated‐GQDs/carboxyl‐GQDs	Prostate cancer	PSA	0.29 pg mL^−1^	[334]
g‐C_3_N_4_/graphene	Lung, head, neck, melanoma, hepatocellular carcinoma	SCCA	8.53 pg mL^−1^	[219]
g‐C_3_N_4_ nanosheets/Au electrode	–	DNA	0.00001 pg mL^−1^	[224]
g‐CNQDs/AuNPs	–	T‐DNA	0.01 fM.	[225]
AuNPs/CQDs‐PEI/GO	–	CEA	1.67 pg mL^−1^	[335]
CQDs coated mesoporous silica nanoparticles	–	MCF‐7 cells	230 cells mL^−1^	[336]
NCQDs‐DNA	–	miRNA‐21	10 aM	[337]
PtNi/CD‐ConA	–	MCF‐7 cells	300 cells mL^−1^	[338]
N‐CQDs	–	AFP	3.3 pg mL^−1^	[339]
CDs	–	CEA, PSA, AFP	3–6 pg mL^−1^	[340]
CDs	–	PSA	0.5 pg mL^−1^	[341]
CQDs	–	CEA	0.00000026 pg mL^−1^	[342]
CdTe/CDs	–	SCCA	0.0063 pg mL^−1^	[343]
Nafionecarbon nanodots nanocomposite films	–	AFP	0.1 pg mL^−1^	[344]
CDs	–	CA125	4.3 mU mL^−1^	[345]

a) Au, gold; Ag, silver; rGO, reduced graphene oxide; GQDs, graphene quantum dots; g‐C_3_N_4_, graphitic carbon nitride; g‐CNQDs, graphitic carbon nitride quantum dots; CQDs, carbon quantum dots; PEI, polyethyleneimine; GO, graphene oxide; NCQDs, nitrogen carbon quantum dots; Pt, platinum; Ni, nickel; CDs, carbon dots; CdTe, cadmium telluride.

### 4.2 Electrochemical Biosensors

The electrochemical biosensor broadly works on the principle of the change in biological signal converting to the electrical signal. This technique is particularly efficient for the protein biomarkers immunoassays due to their size, ease of use, high sensitivity, and cost‐effectiveness.^[^ ^11^ ^]^ The electrochemical biosensor system consists of three major components: 1) A recognition element or bioreceptor that interacts with the analyte; 2) An electrode that works as a signal transducer, which displays a change when the analyte interacts with the recognition element; 3) An electronic system which assists in the data management.^[^ ^231^ ^]^

#### 4.2.1 Graphene‐Based Electrochemical Biosensors

Graphene remains one of the most sought‐after materials because of its strong mechanical strength, flexibility, theoretically high surface area, good electrical, and thermal conductivity.^[^ ^232^ ^]^ Being a 2D carbon allotrope, the 2D surface of graphene forms a base for the adsorption of particular biomolecules. Owing to these merits, graphene has been intensively used for the detection of cancers.^[^ ^233^ ^]^ The sensitivity and selectivity can be finely enhanced using techniques such as oxygen plasma treatment, graphene hybrids formation, heteroatom doping, covalent bonding with dienophiles, and non‐covalent bonding with pyrene derivatives.^[^ ^234^ ^]^ In most cases, graphene behaves as a conductive template to immobilize the bio‐recognition units in the biosensor system.

AuNPs have dominated the biosensor field thanks to their adequate chemical stability, biocompatibility, and high activities, making them excellent scaffolds with graphene for biosensor construction.^[^ ^235^ ^]^ For example, Chen et al. introduced a sandwich‐type electrochemical biosensor using carboxyl graphene nanosheets to detect both CEA and AFP biomarkers concurrently immobilized on a CS‐Au nanoparticles (CHIT‐AuNPs) modified electrode. The sensor exhibited LOD of 0.1 and 0.05 ng mL^−1^ for CEA and AFP, respectively.^[^ ^236^ ^]^ The combination of graphene and AuNPs also increases the electron transport for effectual antibody loading and offers a large surface area for antigen binding.^[^ ^237^ ^]^ By self‐assembly on the GCE, graphene‐conjugated AuNPs were developed for AFP biomarker detection.^[^ ^238^ ^]^ Also, rGO and AuNP composites were developed on the surface of the electrode for impedimetric detection as DNA biosensors, with both complementary and noncomplementary sequences.^[^ ^239^ ^]^ In a study by Bai et al., a label‐free electrochemical biosensor for DNA/miRNA detection was developed utilizing AuNPs‐toluidine blue‐GO nanocomposites, and the biosensor demonstrated an impressive BOD of 2.95 pM.^[^ ^240^ ^]^ Apart from DNA detection, label‐free electrochemical biosensors have also been exploited for miRNA detection. By employing AgNPs in polyaniline and N‐doped graphene, a LOD of miRNA of 0.2 fM has been achieved.^[^ ^241^ ^]^

**Figure 7:**
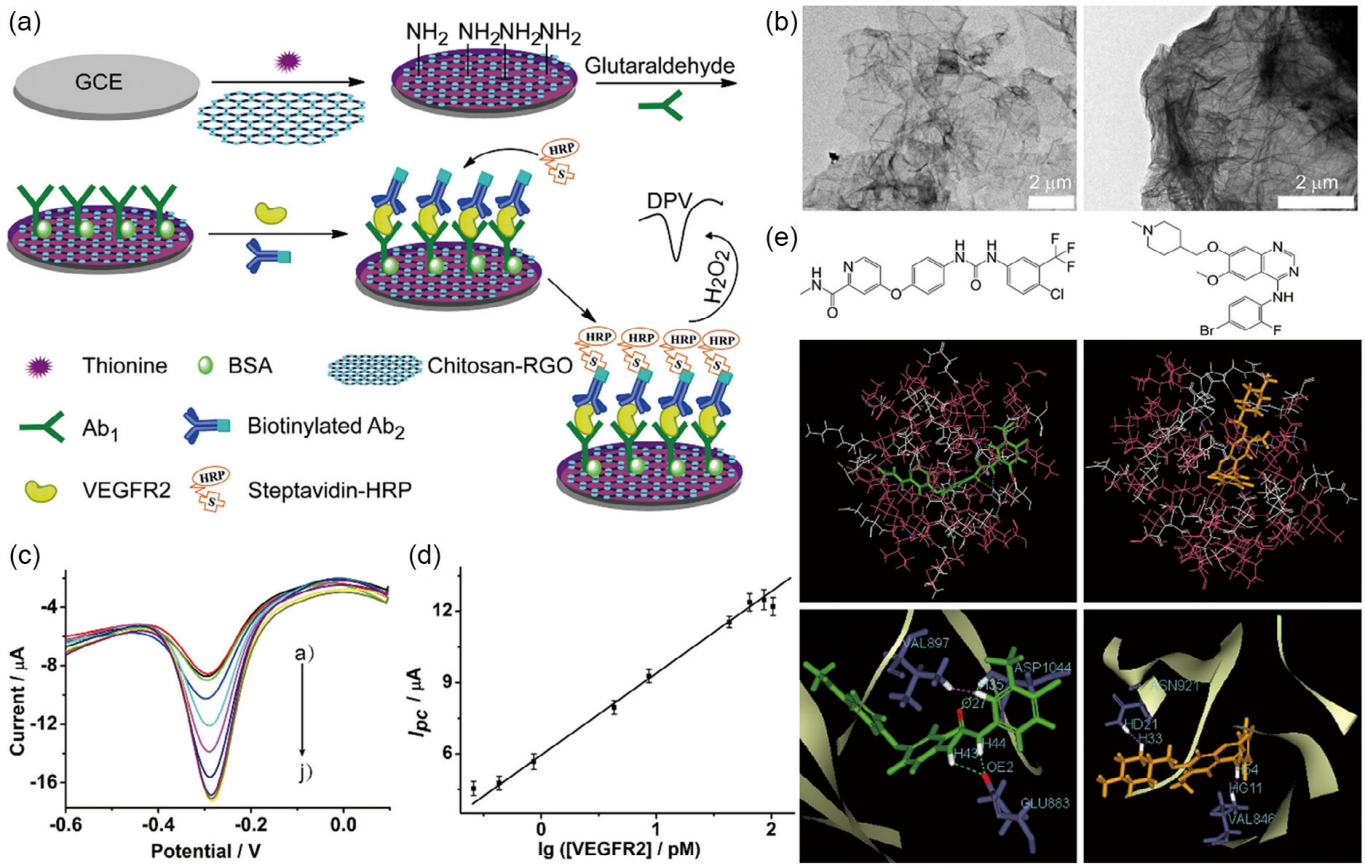
a) The electrochemical biosensing platform designed for protein detection is shown schematically. b) A comparison of the rGO with (left) and without (right) chitosan using TEM images. c) DPV curves for detecting a range of VEGFR2 concentrations, from: a) 0 to b) 0.3, c) 0.4, d) 0.9, e) 4.3, f) 8.6, g) 43, h) 65, i) 86, to j) 103 pM. d) The associated curve of calibration for (c). e) The sorafenib and vandetanib chemical formulas. Below is a representation of the respective binding models for VEGFR2‐sorafenib and VEGFR2‐vandetanib. a–e) Reproduced with permission.^[^ ^242^ ^]^ Copyright 2014, Springer Nature.

In another work, Wei et al. established an electrochemical detection technique for VEGFR2 protein identification.^[^ ^242^ ^]^ This study modified the working electrode with CS‐functionalized rGO to significantly increase the electrical conductivity for detecting VEGFR2 protein by electrochemical biosensing platform (**Figure** **7**a). The effect of CS in RGO was analyzed and confirmed by transmission electron microscope (TEM), as demonstrated in Figure 7b. The rGO without CS encountered significant agglomeration problems, while the chitosan‐rGO sample with a CS membrane enclosing a few layers of graphene nanosheets exhibited no agglomeration. This structural analysis demonstrated that CS plays a crucial role in enabling the aqueous dispersion of rGO through electrostatic interactions. As depicted in Figure 7c, the peak current of pulse voltammetry (DPV) has demonstrated an increase with the ascending VEGFR2 protein concentration from 0.4 to 86 pM. The reduction current peak showed a logarithmic proportionality to the VEGFR2 protein concentration (Figure 7d). The VEGFR2 protein exhibited a wide concentration, from 0.4 to 86 pM, with a high correlation coefficient of 0.999 and an LOD of 0.28 pM for VEGFR2 protein at a signal‐to‐noise ratio of 3. The interaction between the drug and VEGFR2 involves stabilized hydrophobic interactions and polar residues, playing a significant role in drug stabilization through H‐bonds and electrostatic interactions, as illustrated in Figure 7e. Additionally, the interaction between sorafenib and the tyrosine kinase region of VEGFR2 becomes more specific and selective due to the extra hydrogen bonds involved.

Different biomarkers have been successfully detected by graphene‐derived systems. For example, a reusable GO‐modified electrode was fabricated to detect VEGF in the blood sample, which exhibited a low detection of 31.25 pg mL^−1^.^[^ ^243^ ^]^ In this case, GO acts as an efficient carrier for the Avastin antibody, which in turn alters the amperometric signal to detect the analyte. Likewise, Sharafeldin et al. fabricated the electrochemical biosensor by forming GO‐Fe_3_O_4_ nanocomposites to detect PSA and PSMA cancer biomarkers. The GO‐Fe_3_O_4_ hybrid generated electrical signals, enabling the detection of PSA and PSMA biomarkers at LOD of 15 and 4.8 fg mL^−1^, respectively.^[^ ^244^ ^]^

#### 4.2.2 g‐C_3_N_4_‐Based Electrochemical Biosensors

Thanks to the photoactive nature, high surface area, tunable porosity, and facile synthesis nature, highly sensitive electrochemical biosensors have been realized based on g‐C_3_N_4_. Recently, Low et al. fabricated a photoelectrochemical biosensor wherein AuNP and g‐C_3_N_4_ were cast on the indium tin oxide (ITO) electrode surface for recognizing microRNA‐319a.^[^ ^245^ ^]^ The sensing unit consists of anti‐DNA:RNA antibodies as a target analyte biorecognition unit and g‐C3N4 as a photoactive nanomaterial. The developed biosensing system demonstrated outstanding identification sensibility, achieving an LOD of 2.26 fM.^[^ ^246^ ^]^ A similar electrochemical nanobiosensor system based on g‐C_3_N_4_/AuNPs has also been utilized to detect the PSA biomarker with a low detection limit.^[^ ^247^, ^248^ ^]^ In another study, aptamer molecules were cast on the GCE surface with g‐C_3_N_4_/AuNPs, providing high sensitivity and an LOD of 1.6 pg mL^−1^ for the VEGF biomarkers.^[^ ^249^, ^250^ ^]^ In another report, Luo et al. demonstrated AuNPs/hexagonal carbon nitride tubes (HCNT)‐based photoelectrochemical biosensor for HER2 breast cancer biomarker detection. The HER2 aptamer reacts with the phosphate group located on the aptamer with molybdate to form molybdophophate precipitate, which binds to the HCNT surface and inhibits electron transport, resulting in a remarkably LOD of 0.08 pg m^−1^.^[251a]^

The excellent electrochemical properties of g‐C_3_N_4_ enable it in other applications beyond cancer sensing. Organic dyes find widespread application in food preservation, bakery products, and beverages. Tartrazine, an azo dye commonly utilized in commonly consumed foods, has been implicated in causing mutagenic and carcinogenic ailments. Consequently, the need for monitoring these chemicals has become crucial in sensor technology. Karimi et al.^[251b]^ exhibited the straightforward pyrolysis of melamine to fabricate g‐C_3_N_4_ sheets, serving as a sensitive material for detecting tartrazine. A strong catalytic response in the reduction of tartrazine was achieved through the direct adsorption strategy on the g‐C_3_N_4_ nanosheets, examined using CV and DPV techniques. The notable finding from the study indicated that a thin film of graphite/g‐C_3_N_4_ nanosheets could enhance the electrocatalytic effect, while a thick film might impede the response. The sensor's sensitivity, stability, and real‐time applicability were validated using saffron fake powder.^[^ ^252^ ^]^ Phenolic compounds, including phenol, catechol, quercetin (QR), vanillin, and resveratrol, are largely produced as by‐products in various chemical industries such as pharmaceuticals, antioxidants, cosmetics, agricultural pesticides, dyes, leather, petrochemicals, and textiles. Regrettably, these compounds pose significant toxicity to various biological species, human health, and the environment, leading to complications resulting from their degradation. Therefore, the development of electrode materials for monitoring the levels of these phenolic compounds is considered crucial. Selvarajan et al. presented a composite material of g‐C_3_N_4_/NiO for the electrochemical detection of QR. The g‐C_3_N_4_ was synthesized by heating melamine to 550 °C for 4 h, followed by ultrasonication and mixing with nickel chloride to achieve even distribution and form NiO on the g‐C_3_N_4_ nanosheets. The peak current of the electrocatalytic oxidation of QR increased from 0.010 to 250 mM, with LOD of 0.002 mM achieved through DPV analysis. Upon analysis with real samples, the recovery results demonstrated the high feasibility, selectivity, and sensitivity of the sensor electrode material for electrochemical QR sensing. The electrochemical detection of QR was performed using a material consisting of silver nanoparticles deposited on porous ultrathin g‐C_3_N_4_ nanosheets (AgNPs@g‐CN).^[^ ^253^ ^]^

#### 4.2.3 Carbon‐Dot‐Based Electrochemical Biosensors

CDs are utilized as efficient electrochemical biosensors because of their excellent electronic properties, low intrinsic toxicity, high solubility, and good biocompatibility.^[^ ^254^ ^]^ In a typical work, Gao and co‐workers fabricated a CDs‐based electrochemical biosensor using polyamidoamine dendrimers capped‐CDs (PAMAM‐CDs) and Au nanocrystal nanocomposites.^[^ ^255^ ^]^ The amine groups in graphitic PAMAM‐CDs interact with each other to create PAMAM‐CDs/Au nanocrystal composites, enabling AFP detection with an impressively low LOD of 0.025 pg mL^−1^. Furthermore, the impedimetric approach was employed using bimetallic ZrHf metal–organic framework composites with CDs (CDs‐ZrHf‐MOF) to detect HER2 and MCF‐7 breast cancer cells. The LOD were 19 fg mL^−1^ and 23 cells mL^−1^ for HER2 and MCF‐7 cells, respectively.^[^ ^256^, ^257^ ^]^ **Table** **2** summarizes typical electrochemical biosensors performance based on carbon‐based nanomaterials and their detection limits for various biological markers of cancer.

**Table 2:** Electrochemical biosensors produced for cancer biomarkers detection

Carbon systemsa)	Detection methoda)	Cancer types	Biomarkers	LOD	Refs.
GR/AuNPs	CV	–	PSA	590 pg mL^−1^	[346]
GO/Au	DPV	Prostate	VEGF	50 pg mL^−1^	[347]
Graphene/Au/GCE	CV	Breast cancer	MUC1	38 cells mL^−1^	[348]
AuNP‐graphene/GCE	CV	–	CEA	10 pg mL^−1^	[349]
AuNPs/graphene/Au electrode	DPV	–	miR‐ 21	6 fM	[350]
Apt/NH_2_‐graphene‐THI‐AuNPs and PB‐PEDOT AuNPs/SPE	DPV	Lung cancer	CEA	2 pg mL^−1^	[351]
Porous hollowed‐Ag‐Au/Graphene/SPCE	DPV	Prostate cancer	PSA	0.13 pg mL^−1^	[352]
ZrO_2_–rGO/ITO	DPV	Oral cancer	CYFRA‐21‐1	122 pg mL^−1^	[353]
Graphene–Au/GCE	DPV	–	AFP, CEA, CA125, PSA	0.062, 0.048, 0.077 and 0.060 pg mL^−1^	[354]
rGO/AuNPs	EIS	–	CEA	5000 pg mL^−1^	[355]
rGO/GCE/AuNPs	EIS	–	CEA, AFP	1.64, 1.33 pg mL^−1^	[356]
ZrO_2_/rGO	DPV	Oral cancer	CYFRA‐21‐1	10 pg mL^−1^	[357]
Au/GO	DPV	Prostate cancer	PSA	1000 pg mL^−1^	[347]
3D graphene	DPV	–	CEA	90 pg mL^−1^	[358]
Au‐AgNPs/rGO@PDA	CV	–	CEA	0.286 pg mL^−1^	[359]
APTES/MoO_3_/rGO	CV, DPV, EIS	Breast cancer	HER2	1.0 pg mL^−1^	[360]
GCE/GNR/Au	DPV	–	CEA	1.5 pg mL^−1^	[361]
PWE/AuNPs/PANI/rGO/MB, Fc‐COOH	DPV	–	CEA, AFP	0.8, 0.5 pg mL^−1^	[362]
A‐Thi‐CAuNP, ‐Cd^2+^‐CAuNP, DAP‐	SWV	–	PSA, CEA, AFP	4.8, 2.7, 3.1 pg mL^−1^	[363]
rGO/GCE	DPV	Breast cancer	HER2	210 pg mL^−1^	[364]
GQDs‐AuNRs	DPV	Prostate cancer	PSA	140 pg mL^−1^	[365]
g‐C_3_N_4_/ITO‐AuNPs	EIS	Breast cancer	microRNA‐319a	2.26 fM	[246]
HCNT/AuNPs	CV	Breast cancer	HER2	0.08 pg mL^−1^	[251a]
3D graphene/AuNPs‐CuS/rGO	CV and EIS	Lung cancer	CYFRA‐21‐1	100 pg mL^−1^	[366]
3D porous network graphene aerogel	DPV and EIS	Breast cancer	CA 15‐3	0.03 mU mL^−1^	[367]
AgNPs/N‐doped graphene	DPV	Breast cancer	miRNA‐ 21	0.2 fM	[241]

a) GR, graphite; AuNPs, gold nanoparticle; GO, graphene oxide; GCE, glassy carbon electrode; SPEC, screen printed electrode; THI, thionine; ZrO_2_, zirconium oxide; rGO, reduced graphene oxide; ITO, indium tin oxide; PDA, polydiacetylene; APTES, 3‐aminopropyltriethoxysilane; MoO_3_, molybdenum trioxide; GNR, gold nanorod; PWE, paper working electrode; PANI, polyaniline; MB, myoglobin; Fe, iron; COOH, carboxylic acid; GQDs, graphene quantum dots; g‐C_3_N_4_, graphitic carbon nitride; HCNT, hexagonal carbon‐nitrogen tubes; CuS, copper sulfide; N‐doped, nitrogen doped; CAuNp, carbon gold nanoparticle; CD^2+^, cadmium; DAP, 2,3‐diaminophenazine; DPV, differential pulse voltammetry; CV, cyclic voltammetry; EIS, electrochemical impedance spectroscopy.

### 4.3 Fluorescent Biosensors

In recent years, the fluorescent biosensor is becoming increasingly popular as a sensing platform for detecting cancer biomarkers in real samples.^[^ ^258^ ^]^ The fluorescent biosensor is an optically based sensor that measures the changes in analyte concentration through changes in fluorescence. They are mainly separated into two categories: turn‐on and turn‐off, wherein the analyte causes an increase or decrease in the fluorescence of the probe, respectively. Compared to conventional optical sensors based on the change in the optical absorbance with the addition of analytes, fluorescent sensors exhibit minimal interference arising from the analyte itself. Fluorescent biosensors have gained significant importance over the years due to the advent of multiple fluorescent agents, such as carbon QDs, metallic nanoparticles, fluorophores, and polymeric nanoparticles. Fluorescent biosensors are highly user‐friendly due to their quick turn‐around time for the detection of proteins, enzymes, glucose, food toxins, metal ions, and medicine.^[^ ^259^ ^]^ Fluorescent sensing converts the change in the biological recognition system to a change in electrical signals. The fluorescent signal transparency depends upon the usage of fluorescent dyes, fluorescent protein, and fluorescent nanoparticles.^[^ ^260^ ^]^

Fluorescence resonance energy transfer (FRET) involves energy transmission between the donor and acceptor fluorescent molecules. In the excited state, the donor transfers its energy to the acceptor through nonradiative dipole–dipole couplings.^[^ ^261^ ^]^ The energy transfer occurs as the donor chromophore from a higher excitation state transfers the energy to the acceptor via dipole–dipole interactions.^[^ ^262^ ^]^ FRET forms the basis for many fluorescent sensors, in which the change in fluorescence signal is recorded to demonstrate its selectivity with respect to the acceptor molecule. The fluorescent biosensor has been mainly used in the medical field to detect disease‐specific biomarkers, offering an easy and potential diagnosing technique for identifying various cancer biomarkers.^[^ ^263^ ^]^ To date, various fluorescent probes with highly stable photoluminescence, including organic dyes, QDs, and metal ions, are often conjugated with molecules for use as theranostic agents for sensing purposes.^[^ ^264^, ^265^, ^266^ ^]^

#### 4.3.1 Graphene‐Based Fluorescent Biosensors

Even though graphene is not inherently fluorescent, it can exhibit fluorescence with careful modification. In addition, its excellent physical and chemical properties (surface area, mechanical and electrochemical stability, and zero bandgap) can be well exploited as an efficient base for fluorescence detection. Graphene is an efficient signal transducer and increases molecule absorption and sensitivity due to its unique characteristics.^[^ ^240^, ^267^, ^268^ ^]^

Fluorescence quenching encompasses diverse molecular interactions that lead to a reduction in the fluorescence intensity of a sample.^[^ ^269^ ^]^ Graphene can be used efficiently to quench the fluorescence signal in a biosensing platform, making it ideal for reducing the background signal and increasing sensitivity.^[^ ^270^ ^]^ Compared to other molecular quenchers, graphene showed a high quenching efficiency of *d* ^−4^, where d denotes the separation between the fluorophore and the graphene.^[^ ^271^ ^]^ The finely tunable surface of graphene to incorporate extended functionalities empowers it to obtain excellent antigen–antibody specificity. Furthermore, although graphene is inherently not fluorescent, its 0D counterpart, GQDs, are a kind of fluorescent material, and they have the merits of being simple to synthesize, stable, quick to internalize into tissues, and biocompatible.^[^ ^254^ ^]^ By coupling GO with a molecular aptamer beacon probe and employing enzyme‐assisted signal amplification, the detection of VEGF165 and ATP at concentrations of 1 pM and 4 nM, respectively, was successfully achieved. GO serves as a super‐quencher, efficiently reducing the high background signal.^[^ ^272^ ^]^ Shi et al. designed a GQD‐based FRET sensor conjugated with graphene oxide; through careful regulation of their interaction, the synthesized system exhibited an excellent LOD of 75 pM toward DNA.^[^ ^273^ ^]^

#### 4.3.2 g‐C_3_N_4_‐Based Fluorescence Biosensors

g‐C_3_N_4_, as an excellent fluorescent nanoprobe, is able to directly interact with the analyte of interest to quench the fluorescence, so the analyte concentration is subsequently determined.^[^ ^274^ ^]^ Notably, g‐C_3_N_4_ nanosheets have a large surface area, enabling them to bind with DNA targets and multicolor dye‐labeled DNA probes to develop the DNA biosensor.^[^ ^215^ ^]^ In a typical case, the fluorescence quenching ability of g‐C_3_N_4_ assisted in the detection of 15‐mer DNA fragments and 18‐mer DNA fragments, with an LOD of 75 and 62 pM, respectively.^[^ ^275^ ^]^ g‐C_3_N_4_ has not been explored much in the fluorescent sensor route due to the relatively lower fluorescence. However, the nitrogen content, along with rich carbon, can act as a very efficient platform for the donor and acceptor electrons to interact with the analyte in various fields. For example, many g‐CN nano architectures emit blue fluorescence that may potentially disrupt biological self‐fluorescence, leading to a degradation of the actual signal. Acknowledging this, Liu et al. introduced a ratiometric fluorescence aptasensor utilizing phenyl‐doped g‐CN nanosheets, which display robust green fluorescence as an internal reference fluorophore. This system operates through a PET quenching effect on 6‐carboxy‐X‐rhodamine fluorescent dye‐labeled anti‐adenosine aptamer, facilitating the detection of adenosine.^[^ ^276^ ^]^

#### 4.3.3 CDs‐Based Fluorescence Biosensors

CDs are extremely small carbon‐based nanomaterials (usually in a size range of <10 nm), which exhibit excellent fluorescence due to the quantum confinement effect. CDs are signal tags that are frequently used in cancer biomarker detection. CDs have been proven to efficiently label with capturing antibody/primary antibody against MCF‐7 surface protein, which is responsible for breast cancer.^[^ ^30^ ^]^ Incorporating secondary antibody/detecting antibody labeled with magnetic beads permits their magnetic separation to achieve fluorescence emission spectra. In one typical study, CDs‐MnO_2_ composites were used to detect miRNA155 and MCF‐7 breast cancer cells, demonstrating notably low LOD of 0.1 aM and 600 cells mL^−1^, respectively. This was achieved through the incorporation of CDs onto the DNA probe, with MnO_2_ serving as the quenching agent.^[^ ^277^ ^]^ Similarly, Ghadareh et al. constructed a fluorescence biosensor based on CDs conjugated with PAMAM‐Dendrimers/AuNPs.^[^ ^278^ ^]^ The CDs were attached with the CA125 antibody to determine the CA125 biomarker, demonstrating a relatively low LOD of 0.5 fg mL^−1^.^[^ ^279^ ^]^ In another case, Wu fabricated a fluorescence biosensing system using CDs with silica nanoparticles (CD‐SNPs) and fluorescein isothiocyanate (FITC). The antibody was labeled with FITC and used to detect the AFP biomarker at an LOD of 0.317 μg dL^−1^.^[^ ^280^ ^]^ CDs with Au nanocomposites were also used to detect the CA19‐9 pancreatic cancer tumor marker, with notable high sensitivity and selectivity. The anti‐CA19‐9 biomarker was labeled with horseradish peroxidase enzyme in this study and immobilized on the CQDs/Au nanocomposite surface.^[^ ^281^ ^]^

**Figure 8:**
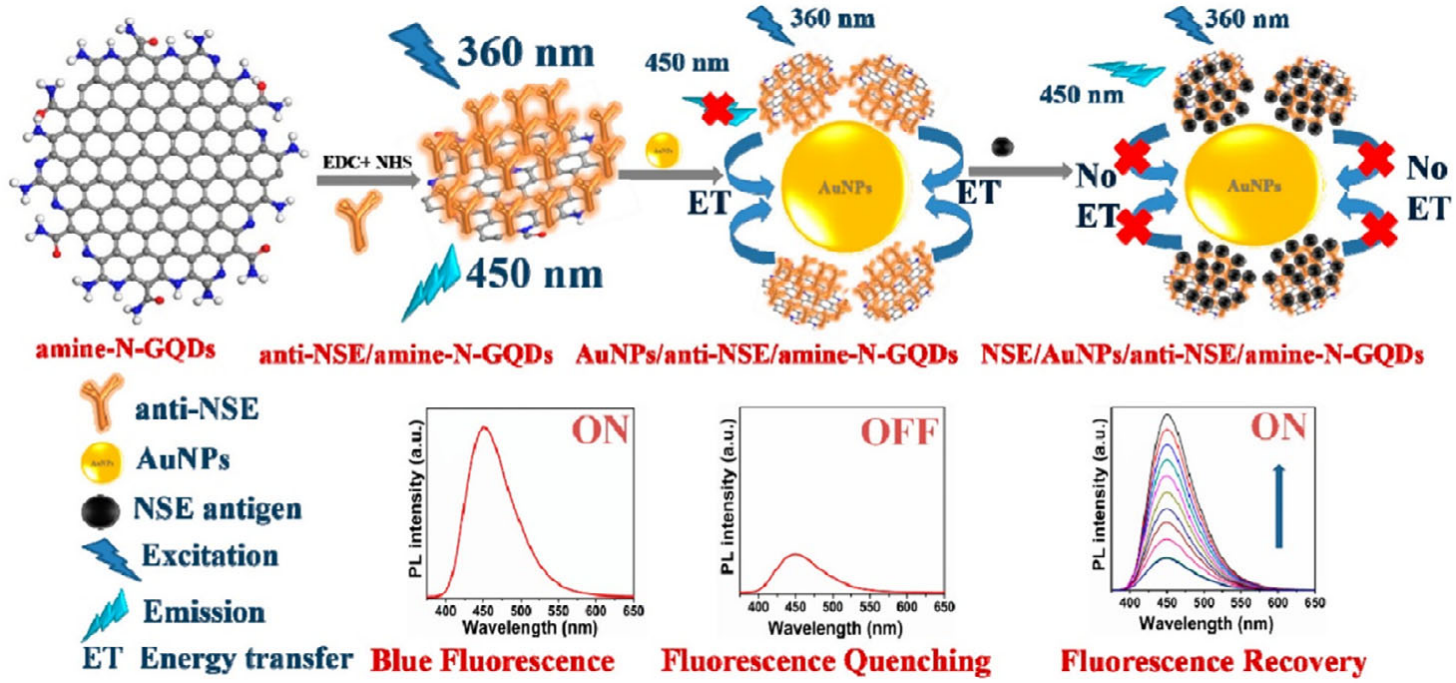
Schematic diagram depicting the operational mechanism of the fluorescent biosensor for detecting small cell lung cancer biomarkers by neuron‐specific enolase antibodies (anti‐NSE)/amine nitrogen‐doped graphene quantum dots with AuNP. Reproduced with permission.^[^ ^282^ ^]^ Copyright 2020, American Chemical Society.

The operational mechanism of fluorescent biosensors based on monoclonal NSE antibody (anti‐NSE)/amine‐N‐GQDs‐AuNPs for detecting small cell lung cancer biomarkers is depicted in **Figure** **8**.^[^ ^282^ ^]^ In this study, amine‐functionalized nitrogen‐doped GQDs were synthesized via a hydrothermal method initiated by citric acid and diethylenetriamine, resulting in an average size of 3 nm. These GQDs were then covalently conjugated with anti‐NSE. The detection of NSE biomarkers relied on the nano surface energy transfer mechanism between AuNPs and the antibody‐conjugated GQDs. In the beginning, the blue emission of the anti‐NSE with the GQDs system experienced quenching (OFF state) due to the presence of highly efficient fluorescent acceptors, AuNPs. However, upon the incorporation of the NSE antigen and hybrid solution, the distance between AuNPs and the conjugated antibody increased, leading to fluorescence restoration (ON state). The resultant anti‐NSE‐AuNPs combination has a very low LOD of 0.09 pg mL^−1^ and a wide linear range between 0.1 pg mL^−1^ and 1000 ng mL^−1^. In addition, this fluorescent biosensing system was an excellent platform for analyzing the biomarkers within human serum samples, yielding a standard recovery value of 94.69%. **Table** **3** enlists the specification and detection limits of fluorescence biosensors developed by carbon‐based materials for various cancer detection.

**Table 3:** Summary of fluorescence biosensors for cancer biomarker detection

Carbon materialsa)	Detection method	Cancer Types	Biomarkers	LOD	Refs.
GQDs‐MoS_2_	FRET	Breast, colorectal, gallbladder, pancreatic, and liver	EpCAM	450 pM	[368]
Ag NPs/GO	Optical	–	PSA	0.23 pg mL^−1^	[369]
Amine‐N‐graphene QDs	FS	Lung cancer	NSE	0.09 g mL^−1^	[282]
g‐C_3_N_4_/AuNPs	FS	–	15‐mer DNA, 18‐mer DNA	75 and 62 pM	[275]
CDs‐aptamer	FRET	Ovarian cancer	CA125	0.0005 pg mL^−1^	[278]
CDs‐AuNPs	FRET	Breast cancer	CA15‐3	0.9 mU mL^−1^	[370]
CQD‐based molecular beacon (MB)	FRET	–	microRNA‐21	0.3 nM	[371]
CD‐doped silica nanoparticles	FS	–	AFP	0.317 mg mL^−1^	[372]
CDs‐AuNPs	FS	–	BRCA1 DNA/RNA	1.5 Nm, 2.1 nM	[373]
CDs	FS	–	Mucin 1	2 nM	[374]
CQDs/Au nanocomposite	FS	Pancreatic cancer	CA 19‐9	0.007 U mL^−1^	[281]
CDs	FS	–	AFP	0.0002 pg mL^−1^	[375]
GO	Optical	–	VGEF	0.25 nM	[249]

a) Ag NPs, silver nanoparticle; GQDs, graphene quantum dots; MoS_2_, molybdenum disulfide; GO, graphene oxide; N‐QDs‐,nitrogen quantum dots; CDs, carbon dots; g‐C_3_N_4_, graphitic carbon nitride; CQDs, carbon quantum dots; MB, myoglobin; FS, fluorescence spectroscopy; FRET, fluorescence resonance energy transfer.

### 4.4 Surface Plasmon Resonance Biosensors

SPR‐based biosensors are considered an attractive biomolecule detection technique due to their numerous important properties.^[^ ^283^ ^]^ First, SPR is a label‐free sensing method that eliminates the need for labeling to generate the signal.^[^ ^284^ ^]^ Additionally, it is capable of detecting the trace amount of desired analytes from composites and analyzing them at sub‐nanogram levels in real‐time.^[^ ^285^ ^]^ Moreover, these sensors are favored for proteome profiles and biomolecule interactions based on affinity toward each other, encompassing phenomena such as the binding of antigens and antibodies, the kinetics of ligand–receptor interactions, enzyme–substrate reactions, and the cartography of epitopes.^[^ ^286^ ^]^ Last but not least, SPR can sense biomolecules with incredibly high selectivity and specificity.^[^ ^287^ ^]^ Based on these advantages, the SPR biosensor is undoubtedly considered a powerful biomedical and clinical analysis tool.^[^ ^286^ ^]^

SPR‐based sensing method allows the arrangement of various transducer formations, such as classical Kretschmann and Otto configurations.^[^ ^288^ ^]^ The classical Kretschmann configuration is the most widely used, which includes SPR imaging,^[^ ^289^ ^]^ nanoparticle‐based LSPR,^[^ ^290^ ^]^ long‐range SPR,^[^ ^291^ ^]^ fiber‐optic sensing,^[^ ^292^ ^]^ and phase sensing.^[^ ^293^ ^]^ The typical structure of an SPR sensor contains a thin metals layer (e.g., Au and Ag) coated on a prism, which separates the prism from the sensing medium, in which the immobilization of analyte on the gold surface material, as well as the binding of the analyte and receptor, takes place on the surface.^[^ ^294^ ^]^

Cancer biomarker sensing via SPR biosensors has been increased due to their high selectivity and sensitivity for some particular analytes.^[^ ^295^ ^]^ Several materials have been assessed for their suitability as the sensing element of the biosensor. Nanomaterials have emerged as a promising option for developing highly sensitive SPR biosensing devices with excellent refractive index sensitivity. Among the various nanomaterials studied, 2D graphene stands out as one of the most attractive nanomaterials due to the unique aforementioned features, such as high surface area, good strength, mechanical flexibility, high optical transparency, and carrier mobility.^[^ ^296^ ^]^ Graphene is an absorbing or binding material with a high surface area, making it an essential component in biosensing systems. Hence, incorporating graphene into a biosensor system effectively enhances the biosensor performance.^[^ ^297^ ^]^

The basic mechanism of the SPR‐based biosensor relies on changes in the material properties or refractive index due to antigen–antibody interaction, which generates different signals at different concentrations.^[^ ^298^ ^]^ SPR is an optical phenomenon that occurs when a metal surface is struck by an incident light photon. Notably, the AuNPs surface has exhibited superior sensing accuracy, primarily influenced by three critical factors, an augmentation in the absolute mass during each binding event, an elevation in the bulk refractive index of the analyte, and plasmon fluctuations creating an electric field between the metal surface and the sample,^[^ ^299^, ^300^ ^]^ facilitating an electromagnetic interaction connecting the localized surface plasmon (LSP) found in metallic nanoparticles with the SPR exhibited by the sensing film.^[^ ^289^, ^301^ ^]^ A graphene layer was applied to the gold surface in order to increase the detection capacity of SPR‐based biosensors. This was accomplished because of the extensive surface area, superior biomolecule adsorption of the graphene, and significant changes in refractive index at the graphene–gold interface.^[^ ^302^ ^]^ The resultant graphene‐based biosensors presented 1 + 0.4 L (in prism waveguide, L represents the quantity of graphene layers) and 1 + 0.45 L (in the planar waveguide) folds with higher sensitivity compared to traditional SPR biosensors.

**Figure 9:**
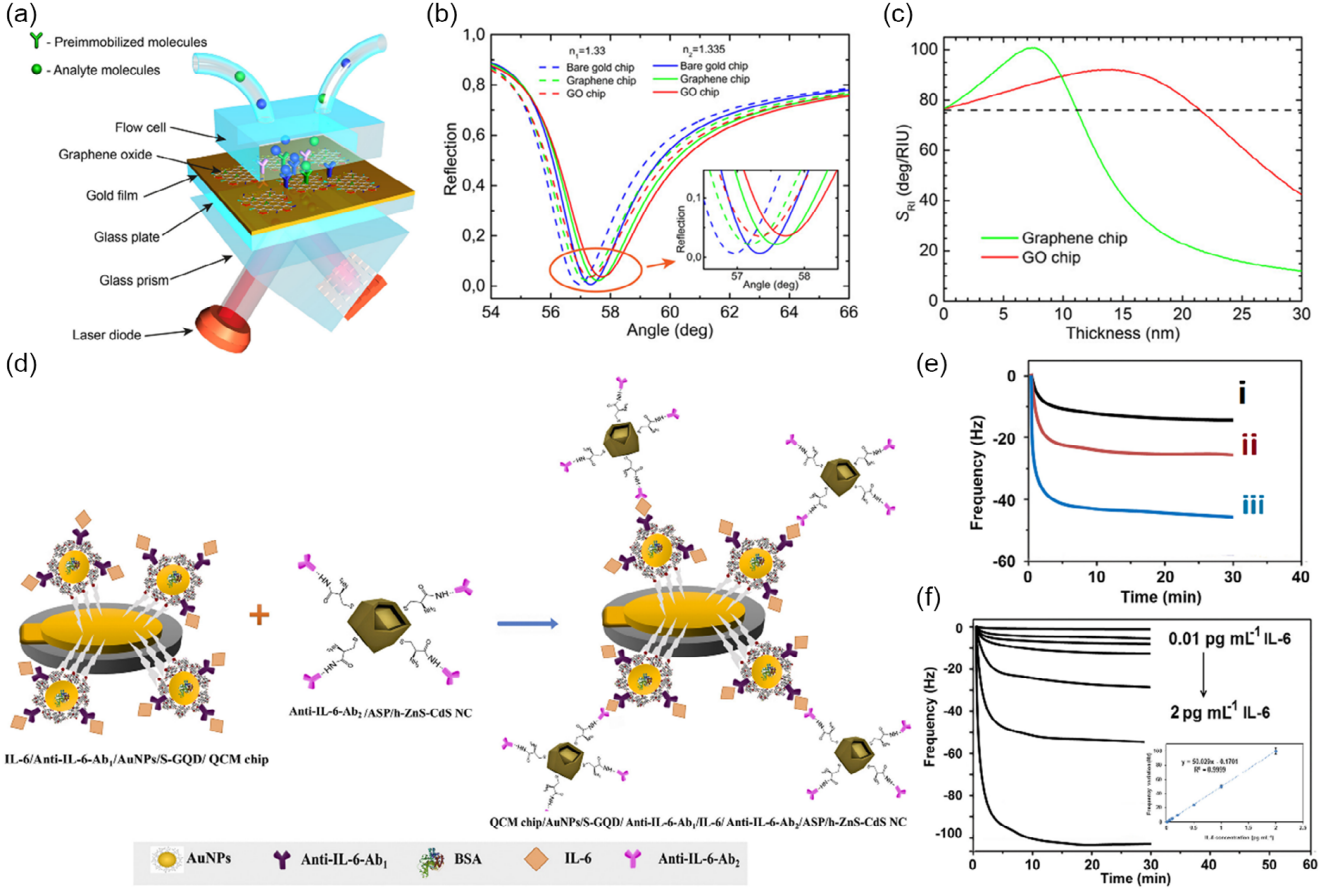
a) Schematic diagram of the SPR biosensor equipped with the GO‐linking layer. b) SPR profiles of the unmodified gold sensor chip, monolayer graphene chip, and GO chip. c) The sensitivity to alterations in the refractive index of the respective SPR biosensor chips. a–c) Reproduced with permission.^[^ ^305^ ^]^ Copyright 2015, American Chemical Society. d) Schematic illustration of QCM‐based immunosensor. e) QCM curves (frequency vs time) of (i) anti‐IL‐6‐Ab1/AuNPs/S‐GQD/QCM, (ii) IL‐6/anti‐IL‐6‐Ab1/AuNPs/S‐GQD/QCM, and (iii) anti‐IL‐6‐Ab2/ASP‐h‐ZnS‐CdS NC conjugated to IL‐6/anti‐IL‐6‐Ab1/AuNPs/S‐GQD/QCM. f) Concentration effect on QCM frequency. The inset shows the corresponding calibration curve. d–f) Reproduced with permission.^[^ ^321^ ^]^ Copyright 2021, Elsevier.

Conventionally, SPR‐based immunosensors have been utilized to detect breast cancer biomarkers like CA15‐3 for over a decade.^[^ ^303^ ^]^ The SPR surface could be further altered by incorporating a nanocomposite of gold and zinc oxide (Au/ZnO) or a traditional gold/chromium (Au/Cr) film to enhance chip performance.^[^ ^304^ ^]^ As for graphene, Stebunov et al. pioneeringly introduced an airbrushing technique to deposit 2D GO on as the linking film to tightly adsorb biomolecules, as displayed in **Figure** **9**a.^[^ ^305^ ^]^ It turns out that the developed airbrushing method realized a tunable and homogeneous GO layer with large surface areas, and its large number of binding sites for the biomolecules ultimately increases the sensitivity. As demonstrated by SPR curves in Figure 9b, there are obvious shifts for the bare gold chip, graphene chips, and GO‐covered chip, and their corresponding dependencies of refractive index changes (SRI) (Figure 9c) unravel 32% and 20% enhanced sensitivity for graphene and GO‐based chips compared to the bare gold chip. Similarly, Chiu et al. demonstrated that adding carboxyl groups could effectively improve and control GO SPR‐based immunoaffinity biosensors through the plasmonic coupling mechanism, achieving a low LOD of 0.01 pg mL^−1^.^[^ ^306^, ^307^ ^]^ In another case, Li et al. proposed a GO‐AuNPs composite‐based SPR biosensor, in which the sensor chip was equipped with DNA‐GO‐AuNPs to identify miRNA and adenosine, achieving a heightened sensitivity with an LOD as low as 0.1 fM.^[^ ^308^ ^]^ Additionally, cytokeratin 19 was detected in a GO‐based SPR sensor for lung cancer diagnosis. Through the functional group on the GO surface, the sensor system fixes large amounts of antibodies, enabling an LOD of 1 fg mL^−1^.^[^ ^240^, ^307^ ^]^

The SPR platform can also be enhanced by incorporating low‐dimensional nanomaterials, such as 2D g‐C_3_N_4_ nanosheets and 0D MoS_2_ QDs combined with CS‐stabilized AuNPs (CS‐AuNPs) placed on the surface.^[^ ^309^ ^]^ The MoS_2_QDs/g‐C_3_N_4_CS‐AuNPs‐based sensor exhibits a strong binding affinity with the PSA biomarker, and it has the highest selectivity and reliability with the lowest LOD of 0.77 ng mL^−1^.^[^ ^310^ ^]^

### 4.5 Quartz Crystal Microbalance Biosensors

QCM biosensors, also known as mass‐based or piezoelectric biosensors, are label‐free mass‐sensitive devices.^[^ ^311^ ^]^ Accurate quantification of biomarker concentrations is possible because the mass of absorbed analytes on the piezoelectric crystal correlates directly with the resonance frequency of the crystals.^[^ ^312^, ^313^ ^]^ Typically, QCM biosensors contain a quartz crystal coated with electrode layers on both the top and bottom sides, and it is attached to an oscillating circuit that applies an external electrical field to the crystals.^[^ ^314^, ^315^, ^316^ ^]^ The presence of any mass deposition on the electrode leads to alterations in the crystal's frequency response, which can be observed through the oscillating circuit system. The QCM surface needs to be modified with receptors that can capture and immobilize target molecules onto the surface of the crystal, and cancer‐causing antigen and antigen‐specific antibodies are used to capture the analyte on the crystal surface and produce a frequency response.^[^ ^46^, ^317^ ^]^ It is well acknowledged that QCM‐based biosensors are one of the most sensitive devices with antigens detection limitations in the picogram range, enabling them as powerful tools for the analysis of tumor marker detection and differentiation between cancer cells and normal cells.^[^ ^318^ ^]^

Carbon‐based nanomaterials are widely used as supporting agents to increase the performance of AuNP‐based QCM biosensors and prevent AuNPs aggregation. These carbon materials are hybridized with AuNPs and coated on the crystal surface. For example, the combination of graphene oxide with AuNPs was developed for CEA detection.^[^ ^319^, ^320^ ^]^ GO‐AuNPs were deposited on the QCM electrode by in situ synthesis method, and anti‐CEA was coated on the surface of the electrode to react with CEA. In this system, GO provides quick transduction of signals from biochemical reactions to electrode outputs, while AuNPs help to immobilize anti‐CEA by forming stable Au–S covalent interactions with the sulfur atom in mercaptoacetic acid. GO‐AuNPs increase the sensitivity of the reaction with a low LOD of 0.09 ng mL^−1^, and the value could further decrease to 0.06 ng mL^−1^ with simply oxygen plasma treatment due to a higher density of AuNPs.

Recently, Atar and Yola demonstrated that nanocomposites consisting of hollow ZnS‐CdS nanocages (h‐ZnS‐CdS NC) and sulfur‐doped GQD functionalized with gold nanoparticles (AuNPs/S‐GQD) provide excellent QCM biosensors for determining the presence of interleukin‐6 (IL‐6), as depicted in Figure 9d.^[^ ^321^ ^]^ Here, S‐GQDs not only prevent AuNPs aggregation but also act as a supporting layer for AuNPs. Due to the amino‐gold affinity, AuNPs/S‐GQD could strongly conjugate with anti‐IL‐6 antibodies, which subsequently reacted with h‐ZnS‐CdS NC in the presence of target IL‐6. In Figure 9e, while sensorgram b of the QCM curves shows a rise in QCM frequencies as an outcome of the immobilization of antigen IL‐6 with anti‐IL‐6‐Ab1 on the surface of QCM gold, sensogram a of the QCM curves shows the starting point for anti‐IL‐6‐Ab1/AuNPs/S‐GQD/QCM, while Sensogram b exhibits an increase in QCM frequencies. This sensitivity is ascribed to the essential and distinct interactions involving IL‐6/anti‐IL‐6‐Ab1/AuNPs/S‐GQD and anti‐IL‐6‐Ab2/ASP‐h‐ZnS‐CdS NC, resulting in heightened frequency fluctuations. The concentration effect of IL‐6 on QCM frequencies was also investigated, as shown in Figure 9f. The developed QCM immunosensor exhibited an impressively low LOD of 3.33 fg mL^−1^, along with a linear range spanning from 0.01 to 2.0 pg mL^−1^. Moreover, it demonstrated excellent storage stability for over eight weeks. In another report, Jandas et al. demonstrated GO/AuNP‐coated QCM sensors utilizing the CEA antibody for precise and real‐time quantification of CEA. The designed biosensor recognizes and captures CEA biomolecules with LOD of 0.06 and 0.09 nm mL^−1^ of CEA. Notably, the invented device's sensitivity may be significantly enhanced after treatment with oxygen plasma as it increased the surface active sites responsible for interacting with the antibody of CEA.^[^ ^319^ ^]^ Despite the numerous merits of carbon‐based QCM biosensors, they have been rarely used for biomarker sensors until now, presumably because of the complication within fabricating a consistent carbon nanomaterial thin layer with excellent friction to transducers, as well as the high cost of electrodes. **Table** **4** compiles the carbon‐material‐based SPR and QCM biosensors that have been developed for the purpose of detecting distinct cancer biomarkers with low LOD, including ssDNA, miRNA, CA15‐3, CEA, interleukin‐6, etc. However, more efforts are still required before this type of biosensor becomes a practical analytical tool.

**Table 4:** Summary of SPR and QCM biosensors for cancer biomarker detection

Carbon materialsa)	Detection method	Cancer types	Biomarkers	LOD	Refs
Graphene	SPR	–	ssDNA	500 aM	[376]
GO/AuNPs	SPR	–	mi RNA	0.1 fM	[308]
GO/AuNPs	QCM	Breast, colon, ovarian, and lung cancer	CEA	90 pg mL^−1^	[319]
AuNPs/S‐GQDs‐(h‐ZnS‐CDs‐NC)	QCM	–	Interleukin‐6	0.00333 pg mL^−1^	[321]

a) Au NPs, gold nanoparticle; GO, graphene oxide; S‐GQDs, sulfur graphene quantum dots; CDs, carbon dots; NC, nitrogen carbon; ZnS, zinc sulfide; SPR, surface plasmon resonance; QCM, quartz crystal microbalance.

## 5 Conclusions and Outlook

Early diagnosis of cancer is the ultimate goal of biosensor technology. A simple and sensitive diagnostic method that needs to detect multiple cancer biomarkers at low concentrations in body fluids is pursued by numerous scientists, while carbon‐nanomaterial‐based biosensors have mostly fulfilled these requirements, with additional advantages including portable, biocompatible, enabling efficient detection, diagnosis, management, and treatment for cancer diseases. Until now, various biosensor technologies based on carbon nanomaterials have been devised to realize early cancer diagnosis, and significant advancements in this research field have been achieved. The efficient analysis of cellular alterations and rapid cancer detection at the onset can ultimately change the strategies of following prognosis and treatment, greatly improving the patient's survival rate. In this review, we first comprehensively reviewed common tumor biomarkers, followed by introducing their corresponding cancers. Later, the development of multiple detection techniques for identifying cancer biomarkers is elaborated in detail, demonstrating excellent detection limits in a broad range of fg mL^−1^ to ng mL^−1^, fast signal transduction, noninvasive detection, real‐time monitoring, biocompatibility, and economic friendly, which position carbon‐based nanomaterials as the next‐generation materials for crafting advanced biosensors capable of highly sensitive cancer biomarker detection.^[^ ^74^ ^]^

Despite the considerable efforts and advancements made in utilizing carbon nanomaterials in biosensor applications, their usage is still in the early stages. To enhance performance and enable future commercialization, further efforts are needed to address the following challenges: 1) Developing a reproducible and economical synthesis approach for oxygen‐free and large‐area carbon nanomaterials with phase purity is crucial, which is also a prerequisite for future large‐scale commercialization. A lot more effort has to be made to explore the single structural phase of the carbon material family rather than mixed‐phase formation in case of reversibly reducing the sensitivity and selectivity of biosensors. 2) Currently, various carbon‐nanomaterial‐based sensing systems have been tested successfully only under laboratory conditions for analyzing the quantification of cancer biomolecules/cells and for clinical applications like imaging and therapeutics. However, cancer biomarker detection in real biological specimens, such as blood plasma, blood serum fluid, saliva, or urine, is much more challenging because these samples are way more complex, with various proteins, ions, and other chemicals able to increase false‐positive responses in assays. Consequently, experiments under realistic conditions with practical device fabrication approaches and operation procedures must be developed for the future reliable applications of these carbon‐based biosensors. 3) Carbon‐based nanomaterials need to be further developed in the biosensing area to realize multiple biomarker analyses at the same time. The diagnosis of specific cancers frequently requires multiplexed analysis. The strategies for concurrently detecting multiple cancer biomarkers or dual‐mode response detection have been demonstrated by other nanomaterials,^[^ ^322^, ^323^ ^]^ but their underlying mechanisms and realization in carbon‐based biosensors are still not well established so far. 4) Even though outstanding achievements have been made, there is still a far distance from commercializing carbon‐based biosensors. For practical clinical use, more investigation should be focused on the long‐term stability and antidisturbance properties of carbon‐based biosensors.

To overcome these challenges, purification and templating methods could be one solution to increase the sensitivity of the sample in the biosensing system, in which the materials’ purities, their specific surface area, and conductivity can be significantly enhanced. Another straightforward approach is to explore new compositions and morphologies of carbon nanomaterials. Carbon‐based nanomaterials could be flexibly hybridized with other functional materials to overcome their intrinsic drawbacks by modifying the structure/morphology and enriching the functionality by virtue of their synergistic effects, thereby enhancing sensing performance. Apart from the carbon nanomaterials introduced in this review, more investigation should focus on designing biosensors based on fullerene materials,^[^ ^324^, ^325^, ^326^, ^327^, ^328^ ^]^ which are observed as promising candidates to identify cancer biomarkers according to recent progress.^[^ ^329^, ^330^ ^]^ It is believed that fullerene‐based sensors are able to use molecularly imprinted polymers and dendrimers as recognition agents rather than biomolecules, which are the starting line in sensing technology and are essential for developing new analyzers.^[^ ^331^ ^]^ Other emerging nanomaterials, like 2D borophene,^[^ ^332^ ^]^ silanol,^[^ ^44^ ^]^ metal nitrides,^[^ ^333^ ^]^ are extremely promising to hybridize with carbon materials for cancer detection. Moving forward, we believe that different families of nanomaterials can make these sensing devices highly potential and allow for rapid, simple, sensitive, and cost‐effective cancer biosensing.

## Conflict of Interest

The authors declare no conflict of interest.

## References

[1] D. Hanahan , Cancer Discov. 2022, 12, 31. 35022204 10.1158/2159-8290.CD-21-1059

[2] R. C. Fitzgerald , A. C. Antoniou , L. Fruk , N. Rosenfeld , Nat. Med. 2022, 28, 666. 35440720 10.1038/s41591-022-01746-x

[3] R. L. Siegel , K. D. Miller , N. S. Wagle , A. Jemal , CA: Cancer J. Clin. 2023, 73, 17. 36633525 10.3322/caac.21763

[4] M. H. Kim , G. Choi , A. Elzatahry , A. Vinu , Y. B. Choy , J. H. Choy , Clays Clay Miner. 2016, 64, 115. 32218609 10.1346/CCMN.2016.0640204 PMC7091641

[5] T. A. Tabish , S. Zhang , P. G. Winyard , Redox Biol. 2018, 15, 34. 29197802 10.1016/j.redox.2017.11.018 PMC5723279

[6] H. Gu , H. Tang , P. Xiong , Z. Zhou , J. Nanomater. 2019, 9, 130. 10.3390/nano9010130 PMC6358776 30669634

[7] N. Bouck , Cancer Cells 1990, 2, 179. 1696827

[8] B. Bohunicky , S. A. Mousa , Nanotechnol. Sci. Appl. 2010, 4, 1. 24198482 10.2147/NSA.S13465 PMC3781701

[9] H. Adami , D. Hunter , D. Trichopoulos , in Cancer Epidemiology, Oxford University Press, Oxford, UK 2008.

[10] A. M. Gown , Mod. Pathol. 2008, 21, S8. 18437174 10.1038/modpathol.2008.34

[11] E. Solhi , M. Hasanzadeh , Biomed. Pharmacother. 2020, 132, 110849. 33068928 10.1016/j.biopha.2020.110849

[12] I. E. Tothill , Semin. Cell Dev. Biol. 2009, 20, 55. 19429492 10.1016/j.semcdb.2009.01.015

[13] H. W. Herr , BJU Int. 2011, 108, 479. 21794063 10.1111/j.1464-410X.2011.10487.x

[14] R. Siegel , D. Naishadham , A. Jemal , CA: Cancer J. Clin. 2012, 62, 283. 22987332 10.3322/caac.21153

[15] F. Bray , J. Ferlay , I. Soerjomataram , R. L. Siegel , L. A. Torre , A. Jemal , CA: Cancer J. Clin. 2018, 68, 394. 30207593 10.3322/caac.21492

[16] V. Gajdosova , L. Lorencova , P. Kasak , J. Tkac , J. Sens. 2020, 20, 4022. 10.3390/s20144022 PMC7412172 32698389

[17] H. T. Trinh , S. Mohanan , D. Radhakrishnan , S. Tiburcius , J. H. Yang , N. M. Verrills , A. Karakoti , A. Vinu , Emergent. Mater. 2021, 4, 1067.

[18] S. Mohanan , C. I. Sathish , K. Ramadass , M. Liang , A. Vinu , Small 2023, 10.1002/smll.202303269. 37386787

[19] S. Tiburcius , K. Krishnan , V. Patel , J. Netherton , C. I. Sathish , J. Weidenhofer , J. H. Yang , N. M. Verrills , A. Karakoti , A. Vinu , Bull. Chem. Soc. Jpn. 2022, 95, 331. 10.1039/d2nr00783e 35441642

[20] S. Tiburcius , K. Krishnan , L. Jose , V. Patel , A. Ghosh , C. I. Sathish , J. Weidenhofer , J. H. Yang , N. M. Verrills , A. Karakoti , A. Vinu , Nanoscale 2022, 14, 6830. 35441642 10.1039/d2nr00783e

[21] D. Radhakrishnan , S. Mohanan , G. Choi , J. H. Choy , S. Tiburcius , H. T. Trinh , S. Bolan , N. Verrills , P. Tanwar , A. Karakoti , A. Vinu , Sci. Technol. Adv. Mater. 2022, 23, 225. 35875329 10.1080/14686996.2022.2052181 PMC9307116

[22] S. Tiburcius , K. Krishnan , J. H. Yang , F. Hashemi , G. Singh , D. Radhakrishnan , H. T. Trinh , N. M. Verrills , A. Karakoti , A. Vinu , Chem. Rec. 2021, 21, 1535. 33320438 10.1002/tcr.202000104

[23] G. Choi , H. Piao , Z. A. Alothman , A. Vinu , C. O. Yun , J. H. Choy , Int. J. Nanomed. 2016, 11, 337. 10.2147/IJN.S95611 PMC4725630 26855572

[24] A. Carbone , E. Vaccher , A. Gloghini , L. Pantanowitz , A. Abayomi , P. de Paoli , S. Franceschi , Nat. Rev. Clin. Oncol. 2014, 11, 223. 24614140 10.1038/nrclinonc.2014.31

[25] N. F. Walker , C. Gan , J. Olsburgh , M. S. Khan , Nat. Rev. Urol. 2014, 11, 383. 24934450 10.1038/nrurol.2014.131

[26] S. S. Laha , N. D. Thorat , G. Singh , C. I. Sathish , J. Yi , A. Dixit , A. Vinu , Small 2022, 18, 2104855. 10.1002/smll.202104855 34874618

[27] A. Pulumati , A. Pulumati , B. S. Dwarakanath , A. Verma , R. V. L. Papineni , Cancer Rep. 2023, 6, e1764. 10.1002/cnr2.1764 PMC9940009 36607830

[28] Y. Wang , X. Liu , C. Chen , Y. Chen , Y. Li , H. Ye , B. Wang , H. Chen , J. Guo , X. Ma , ACS Nano 2022, 16, 180. 35015504 10.1021/acsnano.1c05267

[29] R. Tiwari , K. Lajkosz , M. Berjaoui , Y. Qaoud , M. Kenk , C. Woffendin , P. Caron , C. Guillemette , N. Fleshner , Urol. Oncol. Semin. Orig. Investig. 2022, 40, 193. 10.1016/j.urolonc.2022.03.011 35437219

[30] S. Mittal , H. Kaur , N. Gautam , A. K. Mantha , Biosens. Bioelectron. 2017, 88, 217. 27567264 10.1016/j.bios.2016.08.028

[31] J. Yeong , H. Y. J. Lum , C. B. Teo , B. K. J. Tan , Y. H. Chan , R. Y. K. Tay , J. R.-E. Choo , A. D. Jeyasekharan , Q. H. Miow , L.-H. Loo , Gastric Cancer 2022, 25, 741. 35661944 10.1007/s10120-022-01301-0 PMC9226082

[32] L. Fass , Mol. Oncol. 2008, 2, 115. 19383333 10.1016/j.molonc.2008.04.001 PMC5527766

[33] F. Raza , H. Zafar , X. You , A. Khan , J. Wu , L. Ge , J. Mater. Chem. B 2019, 7, 7639. 31746934 10.1039/c9tb01842e

[34] W. Walter , N. Pfarr , M. Meggendorfer , P. Jost , T. Haferlach , W. Weichert , Semin. Cancer Biol., 2022, 84, 3. 33171257 10.1016/j.semcancer.2020.10.015

[35] J. M. Tettero , S. Freeman , V. Buecklein , A. Venditti , L. Maurillo , W. Kern , R. B. Walter , B. L. Wood , C. Roumier , J. Philippe , B. Denys , J. L. Jorgensen , M. C. Bene , F. Lacombe , A. Plesa , M. L. Guzman , A. Wierzbowska , A. Czyz , L. L. Ngai , A. Schwarzer , C. Bachas , J. Cloos , M. Subklewe , M. Fuering-Buske , F. Buccisano , HemaSphere 2022, 6, e676. 34964040 10.1097/HS9.0000000000000676 PMC8701786

[36] S. Gupta , M. K. Gupta , M. Shabaz , A. Sharma , Front. Physiol. 2022, 13, 952709. 36246115 10.3389/fphys.2022.952709 PMC9563992

[37] L. M. Sholl , D. L. Aisner , T. C. Allen , M. B. Beasley , P. T. Cagle , V. L. Capelozzi , S. Dacic , L. P. Hariri , K. M. Kerr , S. Lantuejoul , M. Mino-Kenudson , K. Raparia , N. Rekhtman , S. Roy-Chowdhuri , E. Thunnissen , M. Tsao , M. Vivero , Y. Yatabe , Arch. Pathol. Lab. Med. 2016, 140, 825. 27195432 10.5858/arpa.2016-0163-SA

[38] L. Guralnik , R. Rozenberg , A. Frenkel , O. Israel , Z. Keidar , J. Nucl. Med. 2015, 56, 518. 25698780 10.2967/jnumed.113.131466

[39] R. de la Rica , M. M. Stevens , Nat. Nanotechnol. 2012, 7, 821. 23103935 10.1038/nnano.2012.186

[40] D. Alberti , M. van't Erve , R. Stefania , M. R. Ruggiero , M. Tapparo , S. G. Crich , S. Aime , Angew. Chem., Int. Ed. 2014, 53, 3488. 10.1002/anie.201310959 24615977

[41] D. J. Reen , Methods Mol. Biol. 1994, 32, 461. 7951745 10.1385/0-89603-268-X:461

[42] F. Peng , Y. Su , Y. Zhong , C. Fan , S. T. Lee , Y. He , Acc. Chem. Res. 2014, 47, 612. 24397270 10.1021/ar400221g

[43] Y. Dai , C. C. Liu , Angew. Chem., Int. Ed. 2019, 58, 12355. 10.1002/anie.201901879 30990933

[44] S. Mohanan , X. Guan , M. Liang , A. Karakoti , A. Vinu , Small 2023, 10.1002/smll.202301113. 36967548

[45] A. Mazzaglia , A. Piperno , Nanomater. 2022, 12, 1597. 10.3390/nano12091597 PMC9105479 35564306

[46] T. Pasinszki , M. Krebsz , T. T. Tung , D. Losic , J. Sens. 2017, 17, 1919. 10.3390/s17081919 PMC5579959 28825646

[47] G. Kothandam , G. Singh , X. Guan , J. M. Lee , K. Ramadass , S. Joseph , M. Benzigar , A. Karakoti , J. Yi , P. Kumar , A. Vinu , Adv. Sci. 2023, 10, 2301045. 10.1002/advs.202301045 PMC10288283 37096838

[48] G. P. Mane , S. N. Talapaneni , K. S. Lakhi , H. Ilbeygi , U. Ravon , K. Al-Bahily , T. Mori , D. H. Park , A. Vinu , Angew. Chem., Int. Ed. 2017, 56, 8481. 10.1002/anie.201702386 28382643

[49] G. P. Mane , D. S. Dhawale , C. Anand , K. Ariga , Q. M. Ji , M. A. Wahab , T. Mori , A. Vinu , J. Mater. Chem. A 2013, 1, 2913.

[50] G. P. Mane , S. N. Talapaneni , C. Anand , S. Varghese , H. Iwai , Q. M. Ji , K. Ariga , T. Mori , A. Vinu , Adv. Funct. Mater. 2012, 22, 3596.

[51] L. Jia , G. P. Mane , C. Anand , D. S. Dhawale , Q. Ji , K. Ariga , A. Vinu , Chem. Commun. 2012, 48, 9029. 10.1039/c2cc33651k 22859219

[52] A. Hirsch , Nat. Mater. 2010, 9, 868. 20966925 10.1038/nmat2885

[53] E. H. L. Falcao , F. Wudl , J. Chem. Technol. Biotechnol. 2007, 82, 524.

[54] M. Safari , A. Moghaddam , A. S. Moghaddam , M. Absalan , B. Kruppke , H. Ruckdaschel , H. A. Khonakdar , Talanta 2023, 258, 124399. 36870153 10.1016/j.talanta.2023.124399

[55] A. Gharehzadehshirazi , M. Zarejousheghani , S. Falahi , Y. Joseph , P. Rahimi , J. Sens. 2023, 23, 1482. 10.3390/s23031482 PMC9919359 36772521

[56] S. Kumar , W. Ahlawat , R. Kumar , N. Dilbaghi , Biosens. Bioelectron. 2015, 70, 498. 25899923 10.1016/j.bios.2015.03.062

[57] A. E. F. Oliveira , A. C. Pereira , L. F. Ferreira , Talanta 2023, 252, 123819. 35973345 10.1016/j.talanta.2022.123819

[58] B. Mohan , S. Kumar , H. Xi , S. Ma , Z. Tao , T. Xing , H. You , Y. Zhang , P. Ren , Biosens. Bioelectron. 2022, 197, 113738. 34740120 10.1016/j.bios.2021.113738

[59] E. Dong , T. Chen , M. Fang , W. Zhu , C. Li , Spectrochim. Acta. A. Mol. Biomol. Spectrosc. 2023, 287, 122064. 36347165 10.1016/j.saa.2022.122064

[60] E. Sobhanie , M. Hosseini , F. Faridbod , M. R. Ganjali , J. Electroanal. Chem. 2023, 932, 117244.

[61] S. Das , R. Devireddy , M. R. Gartia , Biosensors 2023, 13, 396. 36979608 10.3390/bios13030396 PMC10046379

[62] R. A. Alawajji , Z. A. N. Alsudani , A. S. Biris , G. K. Kannarpady , Biosensors 2023, 13, 433. 37185508 10.3390/bios13040433 PMC10136100

[63] U. Bora , A. Sett , D. Singh , Biosens. J. 2013, 2, 1.

[64] A. Lenk , R. G. Ballas , R. Werthschützky , G. Pfeifer , in Electromechanical Systems in Microtechnology and Mechatronics: Electrical, Mechanical and Acoustic Networks, Their Interactions and Applications, Springer Science & Business Media, Berlin/Heidelberg, Germany 2010.

[65] Y. Zhang , D. Yang , L. Weng , L. Wang , Int. J. Mol. Sci. 2013, 14, 15479. 23892596 10.3390/ijms140815479 PMC3759869

[66] V. Gubala , L. F. Harris , A. J. Ricco , M. X. Tan , D. E. Williams , Anal. Chem. 2012, 84, 487. 22221172 10.1021/ac2030199

[67] H. S. Nalwa , RSC Adv. 2020, 10, 30529. 35516069 10.1039/d0ra03183f PMC9056353

[68] S. O. Kelley , C. A. Mirkin , D. R. Walt , R. F. Ismagilov , M. Toner , E. H. Sargent , Nat. Nanotechnol. 2014, 9, 969. 25466541 10.1038/nnano.2014.261 PMC4472305

[69] P. R. Solanki , A. Kaushik , V. V. Agrawal , B. D. Malhotra , NPG Asia Mater. 2011, 3, 17.

[70] M. Song , X. G. Lin , Z. J. Peng , S. B. Xu , L. F. Jin , X. D. Zheng , H. Y. Luo , Front. Mater. 2021, 7, 583739.

[71] B. Seven , M. Bourourou , K. Elouarzaki , J. F. Constant , C. Gondran , M. Holzinger , S. Cosnier , S. Timur , Electrochem. Commun. 2013, 37, 36.

[72] S. Kumar , A. S. Kumar , S. Augustine , S. Yadav , B. K. Yadav , R. P. Chauhan , A. K. Dewan , B. D. Malhotra , Biosens. Bioelectron. 2018, 102, 247. 29153946 10.1016/j.bios.2017.11.004

[73] X. Guan , M. Fawaz , R. Sarkar , C.-H. Lin , Z. Li , Z. Lei , P. D. Nithinraj , P. Kumar , X. Zhang , J.-H. Yang , J. Mater. Chem. A 2023, 11, 12837.

[74] A. Y. S. Tan , N. W. Lo , F. Cheng , M. Zhang , M. T. T. Tan , S. Manickam , K. Muthoosamy , Biosens. Bioelectron. 2023, 219, 114811. 36308836 10.1016/j.bios.2022.114811

[75] Y. B. Yang , X. D. Yang , Y. J. Yang , Q. Yuan , Carbon 2018, 129, 380.

[76] J. Wen , Y. Xu , H. Li , A. Lu , S. Sun , Chem. Commun. 2015, 51, 11346. 10.1039/c5cc02887f 25990681

[77] K. P. Loh , D. Ho , G. N. C. Chiu , D. T. Leong , G. Pastorin , E. K. Chow , Adv. Mater. 2018, 30, e1802368. 30133035 10.1002/adma.201802368

[78] N. Losada-Garcia , I. Rodriguez-Oliva , M. Simovic , D. I. Bezbradica , J. M. Palomo , ACS Omega 2020, 5, 4362. 32175483 10.1021/acsomega.9b04332 PMC7066556

[79] L. A. Al-Ani , M. A. AlSaadi , F. A. Kadir , N. M. Hashim , N. M. Julkapli , W. A. Yehye , Eur. J. Med. Chem. 2017, 139, 349. 28806615 10.1016/j.ejmech.2017.07.036

[80] S. Rodriguez-Mozaz , M. J. Alda , M. P. Marco , D. Barcelo , Talanta 2005, 65, 291. 18969798 10.1016/j.talanta.2004.07.006

[81] S. Su , S. M. Chen , C. H. Fan , Green Energy Environ. 2018, 3, 97.

[82] L. Feng , L. Wu , X. Qu , Adv. Mater. 2013, 25, 168. 23161646 10.1002/adma.201203229

[83] H. M. Ma , D. Wu , Z. T. Cui , Y. Li , Y. Zhang , B. Du , Q. Wei , Anal. Lett. 2013, 46, 1.

[84] B. D. Malhotra , A. Chaubey , Sens. Actuators B 2003, 91, 117.

[85] S. Das , B. Saha , M. Tiwari , D. K. Tiwari , Sens. Diagn. 2023, 2, 268.

[86] S. K. Krishnan , E. Singh , P. Singh , M. Meyyappan , H. S. Nalwa , RSC Adv. 2019, 9, 8778. 35517682 10.1039/c8ra09577a PMC9062009

[87] A. Mohammadpour-Haratbar , S. B. A. Boraei , Y. Zare , K. Y. Rhee , S. J. Park , Biosensors 2023, 13, 80. 36671915 10.3390/bios13010080 PMC9855997

[88] R. M. Alsharabi , S. Rai , H. Y. Mohammed , M. A. Farea , S. Srinivasan , P. S. Saxena , A. Srivastava , Oxf. Open Mater. Sci. 2023, 3, itac013.

[89] M. Pirsaheb , S. Mohammadi , A. Salimi , Trends Anal. Chem., 2019, 115, 83.

[90] X. Guan , Z. Li , X. Geng , Z. Lei , A. Karakoti , T. Wu , P. Kumar , J. Yi , A. Vinu , Small 2023, 19, 2207181. 10.1002/smll.202207181 36693792

[91] M. Pourmadadi , E. Rahmani , M. Rajabzadeh-Khosroshahi , A. Samadi , R. Behzadmehr , A. Rahdar , L. F. R. Ferreira , J. Drug Deliv. Sci. Technol. 2023, 80, 104156.

[92] Y. Kosaki , H. Izawa , S. Ishihara , K. Kawakami , M. Sumita , Y. Tateyama , Q. Ji , V. Krishnan , S. Hishita , Y. Yamauchi , J. P. Hill , A. Vinu , S. Shiratori , K. Ariga , ACS Appl. Mater. Interfaces 2013, 5, 2930. 23574358 10.1021/am400940q

[93] S. Deshmukh , K. Pawar , V. Koli , P. Pachfule , ACS Appl. Bio Mater. 2023, 6, 1339. 10.1021/acsabm.2c01016 37011107

[94] I. Banga , D. C. Poudyal , A. Paul , A. Sardesai , S. Muthukumar , S. Prasad , Biosens. Bioelectron. 2023, 13, 100311.

[95] S. M. Hosseini , J. Mohammadnejad , R. Najafi-Taher , Z. B. Zadeh , M. Tanhaei , S. Ramakrishna , ACS Appl. Bio Mater. 2023, 6, 1323. 10.1021/acsabm.2c01000 36921253

[96] V. Naresh , N. Lee , J. Sens. 2021, 21, 1109. 10.3390/s21041109 PMC7915135 33562639

[97] N. Anzar , R. Hasan , M. Tyagi , N. Yadav , J. Narang , Sens. Int. 2020, 1, 100003.

[98] S. Gupta , C. N. Murthy , C. R. Prabha , Int. J. Biol. Macromol. 2018, 108, 687. 29223757 10.1016/j.ijbiomac.2017.12.038

[99] Z. H. Lin , G. F. Wu , L. Zhao , W. C. Lai , IEEE Nanotechnol. Mag. 2019, 13, 4.

[100] M. Wang , X. Duan , Y. Xu , X. Duan , ACS Nano 2016, 10, 7231. 27403991 10.1021/acsnano.6b03349

[101] W. K. Chee , H. N. Lim , N. M. Huang , I. Harrison , RSC Adv. 2015, 5, 68014-.

[102] N. L. Henry , D. F. Hayes , Mol. Oncol. 2012, 6, 140. 22356776 10.1016/j.molonc.2012.01.010 PMC5528374

[103] E. Badila , C. Japie , D. Bartos , Rom. J. Intern. Med. 2014, 52, 223. 25726624

[104] G. Novelli , C. Ciccacci , P. Borgiani , M. P. Amati , E. Abadie , Clin. Cases Miner. Bone Metab. 2008, 5, 149. 22460999 PMC2781197

[105] W. I. Wan-Ibrahim , V. A. Singh , O. H. Hashim , P. S. Abdul-Rahman , Mol. Med. 2016, 21, 861. 26581086 10.2119/molmed.2015.00183 PMC4818258

[106] L. Y. Xia , Y. N. Tang , J. Zhang , T. Y. Dong , R. X. Zhou , Semin. Cancer. Biol. 2022, 86, 1105. 10.1016/j.semcancer.2021.12.012 34979273

[107] J. M. Carethers , B. H. Jung , Gastroenterology 2015, 149, 1177. 26216840 10.1053/j.gastro.2015.06.047 PMC4589489

[108] F. Counago , F. Lopez-Campos , A. A. Diaz-Gavela , E. Almagro , E. Fenandez-Pascual , I. Henriquez , R. Lozano , E. L. Espinos , A. Gomez-Iturriaga , G. de Velasco , L. M. Q. Franco , I. Rodriguez-Melcon , J. Lopez-Torrecilla , D. E. Spratt , L. L. Guerrero , J. I. Martinez-Salamanca , E. Del Cerro , Cancers 2020, 12, 1550. 32545454 10.3390/cancers12061550 PMC7352850

[109] T. Kunej , J. Obsteter , Z. Pogacar , S. Horvat , G. A. Calin , Crit. Rev. Clin. Lab. Sci. 2014, 51, 344. 25123609 10.3109/10408363.2014.944299

[110] S. Toden , A. Goel , Br. J. Cancer 2022, 126, 351. 35013579 10.1038/s41416-021-01672-8 PMC8810986

[111] M. N. Islam , M. K. Masud , M. H. Haque , M. S. Al Hossain , Y. Yamauchi , N. T. Nguyen , M. J. A. Shiddiky , Small Methods 2017, 1, 1700131.

[112] P. R. Srinivas , M. Verma , Y. Zhao , S. Srivastava , Clin. Chem. 2002, 48, 1160. 12142368

[113] D. Li , D. W. Chan , Expert. Rev. Proteomics 2014, 11, 135. 24646122 10.1586/14789450.2014.897614 PMC4079106

[114] G. De Rubis , S. R. Krishnan , M. Bebawy , Trends Pharmacol. Sci. 2019, 40, 172. 30736982 10.1016/j.tips.2019.01.006

[115] R. A. Frank , D. Galasko , H. Hampel , J. Hardy , M. J. de Leon , P. D. Mehta , J. Rogers , E. Siemers , J. Q. Trojanowski , Neurobiol. Aging 2003, 24, 521. 12714109 10.1016/s0197-4580(03)00002-2

[116] M. Sioud , Methods Mol. Biol. 2007, 360, 1. 17172722 10.1385/1-59745-165-7:1

[117] J. Glowska-Ciemny , M. Szymanski , A. Kuszerska , Z. Malewski , C. von Kaisenberg , R. Kocylowski , Int. J. Mol. Sci. 2023, 24, 2539. 36768863 10.3390/ijms24032539 PMC9917199

[118] G. Manasa , R. J. Mascarenhas , S. J. Malode , N. P. Shetti , Biosens. Bioelectron, 2022, 100189.

[119] I. K. Oktaviyanti , D. S. Ali , S. A. Awadh , M. J. C. Opulencia , S. Yusupov , R. Dias , F. Alsaikhan , M. M. Mohammed , H. Sharma , Y. F. Mustafa , M. M. Saleh , Anal. Bioanal. Chem. 2023, 415, 367. 35641643 10.1007/s00216-022-04150-z

[120] H. Cho , C. K. Oh , J. Cha , J. I. Chung , S. S. Byun , S. K. Hong , J. S. Chung , K. H. Han , Prostate Int. 2022, 10, 14. 35229001 10.1016/j.prnil.2022.01.002 PMC8844604

[121] K. Hashimoto , S. Nishimura , T. Ito , N. Oka , R. Kakinoki , M. Akagi , Eur. J. Histochem. 2022, 66, 3377. 35736245 10.4081/ejh.2022.3377 PMC9251608

[122] R. Guo , W. Wu , Gen. Chem. 2016, 2, 89.

[123] A. M. Hazazi , *Ph.D. Thesis,* Prifysgol Bangor University 2017.

[124] J. H. Schieving , M. de Vries , J. M. van Vugt , C. Weemaes , M. van Deuren , J. Nicolai , R. A. Wevers , M. A. Willemsen , Eur. J. Paediatr. Neurol. 2014, 18, 243. 24120489 10.1016/j.ejpn.2013.09.003

[125] P. R. Galle , F. Foerster , M. Kudo , S. L. Chan , J. M. Llovet , S. Qin , W. R. Schelman , S. Chintharlapalli , P. B. Abada , M. Sherman , A. X. Zhu , Liver Int. 2019, 39, 2214. 31436873 10.1111/liv.14223

[126] Y. He , H. Lu , L. Zhang , Prog. Mol. Biol. Transl. Sci. 2019, 162, 199. 30905450 10.1016/bs.pmbts.2019.01.001

[127] J. Y. Choi , S. W. Jung , H. Y. Kim , M. Kim , Y. Kim , D. G. Kim , E. J. Oh , World J. Gastroenterology 2013, 19, 339. 10.3748/wjg.v19.i3.339 PMC3554817 23372355

[128] S. K. Asrani , H. Devarbhavi , J. Eaton , P. S. Kamath , J. Hepatol. 2019, 70, 151. 30266282 10.1016/j.jhep.2018.09.014

[129] A. M. Kabel , A. H. Al-shehri , B. S. Madani , S. I. Al-Shafie , S. A. Amasha , J. Cancer Res. Treat. 2016, 4, 80.

[130] S. Hammarström , Semin. Cancer Biol. 1999, 9, 67. 10202129 10.1006/scbi.1998.0119

[131] M. J. Goldstein , E. P. Mitchell , Cancer Invest. 2005, 23, 338. 16100946 10.1081/cnv-58878

[132] J. M. Jessup , R. Giavazzi , D. Campbell , K. Cleary , K. Morikawa , I. J. Fidler , Cancer Res. 1988, 48, 1689. 3345536

[133] M. Grunnet , J. B. Sorensen , Lung Cancer 2012, 76, 138. 22153832 10.1016/j.lungcan.2011.11.012

[134] R. D. Rosen , A. Sapra , in TNM Classification, StatPearls Publishing, St. Petersburg, FL 2022. 31985980

[135] L. A. Liotta , Sci. Am. 1992, 266, 54. 10.1038/scientificamerican0292-54 1373003

[136] V. V. Levenson , Biochim. Biophys. Acta-Gen. Subj. 2007, 1770, 847. 10.1016/j.bbagen.2007.01.017 17368950

[137] P. R. Srinivas , B. S. Kramer , S. Srivastava , Lancet Oncol. 2001, 2, 698. 11902541 10.1016/S1470-2045(01)00560-5

[138] J. L. Jones , *Ph.D. Thesis*, University of Leicester 1999.

[139] H. Sung , J. Ferlay , R. L. Siegel , M. Laversanne , I. Soerjomataram , A. Jemal , F. Bray , CA: Cancer J. Clin. 2021, 71, 209. 33538338 10.3322/caac.21660

[140] O. I. Olopade , T. A. Grushko , R. Nanda , D. Huo , Clin. Cancer Res. 2008, 14, 7988. 19088015 10.1158/1078-0432.CCR-08-1211 PMC4535810

[141] L. J. van 't Veer , H. Dai , M. J. van de Vijver , Y. D. He , A. A. Hart , M. Mao , H. L. Peterse , K. van der Kooy , M. J. Marton , A. T. Witteveen , G. J. Schreiber , R. M. Kerkhoven , C. Roberts , P. S. Linsley , R. Bernards , S. H. Friend , Nature 2002, 415, 530. 11823860 10.1038/415530a

[142] S. Staff , N. N. Nupponen , A. Borg , J. J. Isola , M. M. Tanner , Genes Chromosom. Cancer 2000, 28, 432. 10862052 10.1002/1098-2264(200008)28:4<432::aid-gcc9>3.0.co;2-j

[143] S. N. Powell , L. A. Kachnic , Oncogene 2003, 22, 5784. 12947386 10.1038/sj.onc.1206678

[144] E. Le Rhun , A. Kramar , S. Salingue , M. Girot , I. Rodrigues , A. Mailliez , F. Zairi , E. Bakhache , Y. M. Robin , S. Taillibert , F. Dubois , J. Bonneterre , M. C. Chamberlain , J. Neurooncol. 2014, 117, 117. 24469852 10.1007/s11060-014-1361-1

[145] S. Yadav , D. R. Mathur , D. S. Singh , Int. J. Clin. Diagn. Pathol. 2021, 4, 99.

[146] A. M. Kabel , J. Oncol. Sci. 2017, 3, 5.

[147] J. T. Gohring , P. S. Dale , X. D. Fan , Sens. Actuators, B Chem. 2010, 146, 226.

[148] R. C. Marques , S. Viswanathan , H. P. Nouws , C. Delerue-Matos , M. B. Gonzalez-Garcia , Talanta 2014, 129, 594. 25127638 10.1016/j.talanta.2014.06.035

[149] N. Iqbal , N. Iqbal , Mol. Biol. Int. 2014, 852748. 25276427 10.1155/2014/852748 PMC4170925

[150] M. Hoffmann , J. Gogola , A. Ptak , Horm. Cancer 2018, 9, 166. 29603059 10.1007/s12672-018-0331-z PMC5945719

[151] R. Ali-Fehmi , E. Abdulfatah , in Ovarian Cancer Immunotherapy (Eds: S. A. Farghaly ), Oxford University Press, Oxford, UK 2018, Ch. 2.

[152] S. Sarojini , A. Tamir , H. Lim , S. Li , S. Zhang , A. Goy , A. Pecora , K. S. Suh , J. Oncol. 2012, 709049. 23319948 10.1155/2012/709049 PMC3540796

[153] J. M. Cragun , Cancer Control 2011, 18, 16. 21273976 10.1177/107327481101800103

[154] P. M. Gocze , D. A. Freeman , J. Am. Med. Assoc. 1993, 269, 3106. 8505808

[155] E. Gasiorowska , T. Kluz , D. Lipski , W. Warchol , A. Tykarski , E. Nowak-Markwitz , Dis. Markers 2019, 3890906. 31583027 10.1155/2019/3890906 PMC6754914

[156] R. Srinivasan , C. E. Gillett , D. M. Barnes , W. J. Gullick , Cancer Res. 2000, 60, 1483-. 10749108

[157] L. M. Gilmour , K. G. Macleod , A. McCaig , W. J. Gullick , J. F. Smyth , S. P. Langdon , Cancer Res. 2001, 61, 2169. 11280782

[158] E. Anastasi , G. G. Marchei , V. Viggiani , G. Gennarini , L. Frati , M. G. Reale , Tumor Biol. 2010, 31, 113. 10.1007/s13277-009-0015-y 20358424

[159] J. H. Kim , S. J. Skates , T. Uede , K. K. Wong , J. O. Schorge , C. M. Feltmate , R. S. Berkowitz , D. W. Cramer , S. C. Mok , J. Am. Med. Assoc. 2002, 287, 1671. 10.1001/jama.287.13.1671 11926891

[160] S. K. Ramaiah , S. Rittling , Toxicol. Sci. 2008, 103, 4. 17890765 10.1093/toxsci/kfm246

[161] N. Ferrara , Trends Cardiovasc. Med. 1993, 3, 244. 21244915 10.1016/1050-1738(93)90046-9

[162] F. Ghavamipour , H. Rahmani , M. Shanehsaz , K. Khajeh , M. Mirshahi , R. H. Sajedi , J. Nanobiotechnol. 2020, 18, 93. 10.1186/s12951-020-00648-9 PMC7302009 32552818

[163] B. Z. Stanger , M. Hebrok , Gastroenterology 2013, 144, 1170. 23622126 10.1053/j.gastro.2013.01.074 PMC3639438

[164] M. E. Jean , A. M. Lowy , D. B. Evans , Gastrointest. Oncol. 2003, 336.

[165] S. Serafini , G. Da Dalt , G. Pozza , S. Blandamura , M. Valmasoni , S. Merigliano , C. Sperti , World J. Surg. Oncol. 2017, 15, 93. 28464920 10.1186/s12957-017-1157-9 PMC5414360

[166] K. S. Goonetilleke , A. K. Siriwardena , Eur. J. Surg. Oncol. 2007, 33, 266. 17097848 10.1016/j.ejso.2006.10.004

[167] R. S. O’Neill , A. Stoita , World J. Gastroenterol. 2021, 27, 4045. 34326612 10.3748/wjg.v27.i26.4045 PMC8311531

[168] J. Song , L. J. Sokoll , J. J. Pasay , A. L. Rubin , H. Li , D. M. Bach , D. W. Chan , Z. Zhang , Cancer Epidemiol. Biomarkers Prev. 2019, 28, 174. 30333219 10.1158/1055-9965.EPI-18-0483 PMC6324978

[169] A. Thapa , A. C. Soares , J. C. Soares , I. T. Awan , D. Volpati , M. E. Melendez , J. Fregnani , A. L. Carvalho , O. N. Oliveira Jr. , ACS Appl. Mater. Interfaces 2017, 9, 25878. 28696659 10.1021/acsami.7b07384

[170] L. Qian , Q. Li , K. Baryeh , W. Qiu , K. Li , J. Zhang , Q. Yu , D. Xu , W. Liu , R. E. Brand , X. Zhang , W. Chen , G. Liu , Transl. Res. 2019, 213, 67. 31442419 10.1016/j.trsl.2019.08.002

[171] N. Bardeesy , R. A. DePinho , Nat. Rev. Cancer 2002, 2, 897. 12459728 10.1038/nrc949

[172] S. Moradi , M. K. Kelarijani , V. Shokri , Cent. Asian J. Med. Pharm. Sci. Innov. 2021, 1, 143.

[173] T. P. Yeo , R. H. Hruban , S. D. Leach , R. E. Wilentz , T. A. Sohn , S. E. Kern , C. A. Iacobuzio-Donahue , A. Maitra , M. Goggins , M. I. Canto , R. A. Abrams , D. Laheru , E. M. Jaffee , M. Hidalgo , C. J. Yeo , Curr. Probl. Cancer 2002, 26, 176. 12399802 10.1067/mcn.2002.129579

[174] M. Rigau , M. Olivan , M. Garcia , T. Sequeiros , M. Montes , E. Colas , M. Llaurado , J. Planas , I. Torres , J. Morote , C. Cooper , J. Reventos , J. Clark , A. Doll , Int. J. Mol. Sci. 2013, 14, 12620. 23774836 10.3390/ijms140612620 PMC3709804

[175] G. Lucarelli , M. Fanelli , A. M. Larocca , C. A. Germinario , M. Rutigliano , A. Vavallo , F. P. Selvaggi , C. Bettocchi , M. Battaglia , P. Ditonno , Prostate 2012, 72, 1611. 22430630 10.1002/pros.22514

[176] T. Bhavsar , P. McCue , R. Birbe , Semin. Oncol. 2013, 40, 259. 23806492 10.1053/j.seminoncol.2013.04.002

[177] R. A. Ghossein , J. Rosai , Cancer 1996, 78, 10. 8646704 10.1002/(SICI)1097-0142(19960701)78:1<10::AID-CNCR3>3.0.CO;2-L

[178] E. S. Leman , R. H. Getzenberg , in Therapeutic Strategies in Prostate Cancer, Clinical Publishing, Oxford, UK 2007, Ch. 2.

[179] T. Vavala , M. Rigney , M. L. Reale , S. Novello , J. C. King , Respirology 2020, 25, 24. 33124087 10.1111/resp.13965

[180] R. Molina , J. M. Auge , X. Bosch , J. M. Escudero , N. Vinolas , R. Marrades , J. Ramirez , E. Carcereny , X. Filella , Tumor Biol. 2009, 30, 121. 10.1159/000224628 19506400

[181] S. A. Ahrendt , J. T. Chow , S. C. Yang , L. Wu , M. J. Zhang , J. Jen , D. Sidransky , Cancer Res. 2000, 60, 3155. 10866304

[182] A. Khanmohammadi , A. Aghaie , E. Vahedi , A. Qazvini , M. Ghanei , A. Afkhami , A. Hajian , H. Bagheri , Talanta 2020, 206, 120251. 31514848 10.1016/j.talanta.2019.120251

[183] H. I , J. Y. Cho, Adv. Clin. Chem. 2015, 72, 107. 26471082 10.1016/bs.acc.2015.07.003

[184] J. Schneider , Adv. Clin. Chem. 2006, 42, 1. 17131623 10.1016/s0065-2423(06)42001-1

[185] K. Okamura , K. Takayama , M. Izumi , T. Harada , K. Furuyama , Y. Nakanishi , Lung Cancer 2013, 80, 45. 23352032 10.1016/j.lungcan.2013.01.002

[186] S. I. Kaya , G. Ozcelikay , F. Mollarasouli , N. K. Bakirhan , S. A. Ozkan , Sens. Actuators B 2022, 351, 130856.

[187] T. N. Zamay , G. S. Zamay , O. S. Kolovskaya , R. A. Zukov , M. M. Petrova , A. Gargaun , M. V. Berezovski , A. S. Kichkailo , Cancers 2017, 9, 155. 29137182 10.3390/cancers9110155 PMC5704173

[188] V. Doseeva , T. Colpitts , G. Gao , J. Woodcock , V. Knezevic , J. Transl. Med. 2015, 13, 55. 25880432 10.1186/s12967-015-0419-y PMC4335536

[189] A. Touhami , J. Nanomed. 2014, 15, 374.

[190] M. E. Karim , in Handbook of Cell Biosensors, Springer, Berlin, Germany, 2021.

[191] V. Perumal , U. Hashim , J. Appl. Biomed. 2014, 12, 1.

[192] E. K. Wujcik , H. Wei , X. Zhang , J. Guo , X. Yan , N. Sutrave , S. Wei , Z. Guo , RSC Adv. 2014, 4, 43725.

[193] Z. Rahmati , M. Roushani , H. Hosseini , Talanta 2022, 237, 122924. 34736661 10.1016/j.talanta.2021.122924

[194] A. Fiorani , G. Valenti , M. Iurlo , M. Marcaccio , F. Paolucci , Curr. Opin. Electrochem. 2018, 8, 31.

[195] C. Ma , Y. Cao , X. Gou , J. J. Zhu , Anal. Chem. 2020, 92, 431. 31679341 10.1021/acs.analchem.9b04947

[196] R. J. Forster , P. Bertoncello , T. E. Keyes , Annu. Rev. Anal. Chem. 2009, 2, 359. 10.1146/annurev-anchem-060908-155305 20636067

[197] W. Y. Zhao , Y. Ma , J. S. Ye , J. Electroanal. Chem. 2021, 888, 115215.

[198] K. Chu , J. R. Adsetts , S. He , Z. Zhan , L. Yang , J. M. Wong , D. A. Love , Z. Ding , Chem.-Eur. J 2020, 26, 15892. 32780915 10.1002/chem.202003395

[199] Y. Du , S. Dong , Anal. Chem. 2017, 89, 189. 28105831 10.1021/acs.analchem.6b04190

[200] S. Zhang , F. L. Rong , C. A. P. Guo , F. H. Duan , L. H. He , M. H. Wang , Z. H. Zhang , M. M. Kang , M. Du , Coord. Chem. Rev. 2021, 439, 213948.

[201] Y. N. Khonsari , S. Sun , Chem. Commun. 2017, 53, 9042. 10.1039/c7cc04300g 28759057

[202] B. Babamiri , D. Bahari , A. Salimi , Biosens. Bioelectron. 2019, 142, 111530. 31398687 10.1016/j.bios.2019.111530

[203] K. Nekoueian , M. Amiri , M. Sillanpaa , F. Marken , R. Boukherroub , S. Szunerits , Chem. Soc. Rev. 2019, 48, 4281. 31215906 10.1039/c8cs00445e

[204] B. Pérez-López , A. Merkoçi , Mikrochim. Acta 2012, 179, 1.

[205] C. Zhang , P. Miao , M. Sun , M. Yan , H. Liu , Small 2019, 15, 1901867. 10.1002/smll.201901867 31379135

[206] Q. Xu , H. Jia , X. Duan , L. Lu , Q. Tian , S. Chen , J. Xu , F. Jiang , Inorg. Chem. Commun. 2020, 119, 108122.

[207] J. Pena-Bahamonde , H. N. Nguyen , S. K. Fanourakis , D. F. Rodrigues , J. Nanobiotechnol. 2018, 16, 75. 10.1186/s12951-018-0400-z PMC6150956 30243292

[208] B. Xing , W. Zhu , X. Zheng , Y. Zhu , Q. Wei , D. Wu , Sens. Actuators B 2018, 265, 403.

[209] S. Xu , Y. Liu , T. Wang , J. Li , Anal. Chem. 2011, 83, 3817. 21513282 10.1021/ac200237j

[210] Y. Zhang , L. Li , H. M. Yang , Y. N. Ding , M. Su , J. T. Zhu , M. Yan , J. H. Yu , X. R. Song , RSC Adv. 2013, 3, 14701.

[211] T. Wang , S. Zhang , C. Mao , J. Song , H. Niu , B. Jin , Y. Tian , Biosens. Bioelectron. 2012, 31, 369. 22099955 10.1016/j.bios.2011.10.048

[212] M. R. Cui , R. Z. Yu , X. M. Wang , H. Zhou , J. Liu , S. S. Zhang , J. Electroanal. Chem. 2016, 781, 410.

[213] A. Mohammadinejad , R. K. Oskuee , R. Eivazzadeh-Keihan , M. Rezayi , B. Baradaran , A. Maleki , M. Hashemzaei , A. Mokhtarzadeh , M. de la Guardia , TrAc-Trends Anal. Chem. 2020, 130, 115961.

[214] H. Nasrollahpour , I. Isildak , M. R. Rashidi , E. A. Hashemi , A. Naseri , B. Khalilzadeh , Cancer Nanotechnol. 2021, 12, 10.

[215] Y. Wang , R. Zhang , Z. Y. Zhang , J. L. Cao , T. Y. Ma , Adv. Mater. Interfaces 2019, 6, 1901429.

[216] A. Wang , C. Wang , L. Fu , W. Wong-Ng , Y. Lan , Nano-Micro Lett. 2017, 9, 47. 10.1007/s40820-017-0148-2 PMC6199047 30393742

[217] E. M. Gross , S. S. Maddipati , S. M. Snyder , Bioanalysis 2016, 8, 2071. 27611228 10.4155/bio-2016-0178 PMC5041308

[218] K. Y. Tomizaki , K. Usui , H. Mihara , Chembiochem 2005, 6, 782. 15791688 10.1002/cbic.200400232

[219] X. Li , X. Zhang , H. Ma , D. Wu , Y. Zhang , B. Du , Q. Wei , Biosens. Bioelectron. 2014, 55, 330. 24412767 10.1016/j.bios.2013.12.039

[220] L. Wu , Y. Hu , Y. Sha , W. Li , T. Yan , S. Wang , X. Li , Z. Guo , J. Zhou , X. Su , Talanta 2016, 160, 247. 27591611 10.1016/j.talanta.2016.07.023

[221] H. J. Song , L. C. Zhang , Y. Y. Su , Y. Lv , J. Anal. Test. 2017, 1, 274.

[222] R. Zou , X. Teng , Y. J. Lin , C. Lu , TrAc-Trends Anal. Chem. 2020, 132, 116054.

[223] L. Wu , Y. Sha , W. Li , S. Wang , Z. Guo , J. Zhou , X. Su , X. Jiang , Sens. Actuators B 2016, 226, 62.

[224] A. O. Idris , E. O. Oseghe , T. A. M. Msagati , A. T. Kuvarega , U. Feleni , B. Mamba , Sensors 2020, 20, 5743. 33050361 10.3390/s20205743 PMC7600177

[225] Z. M. Liu , X. Zhang , X. G. Ge , L. Q. Hu , Y. J. Hu , Sens. Actuators, B 2019, 297, 126790.

[226] K. Shao , B. Wang , A. Nie , S. Ye , J. Ma , Z. Li , Z. Lv , H. Han , Biosens. Bioelectron. 2018, 118, 160. 30075386 10.1016/j.bios.2018.07.029

[227] X. Zhong , X. Li , Y. Zhuo , Y. Q. Chai , R. Yuan , Sens. Actuators, B 2020, 305, 127490.

[228] Y. Sun , C. Huang , X. Sun , Q. Wang , P. Zhao , S. Ge , J. Yu , Microchim. Acta 2021, 188, 353. 10.1007/s00604-021-04962-3 34568991

[229] Q. Zhang , Y. Liu , Y. Nie , Y. Liu , Q. Ma , Anal. Chem. 2019, 91, 13780. 31590487 10.1021/acs.analchem.9b03212

[230] J. Yang , Q. Xia , L. Guo , F. Luo , Y. Dong , B. Qiu , Z. Lin , Chem. Commun. 2020, 56, 6692. 10.1039/c9cc09706f 32412030

[231] A. Hashem , M. A. M. Hossain , A. R. Marlinda , M. A. Mamun , S. Sagadevan , Z. Shahnavaz , K. Simarani , M. R. Johan , Crit. Rev. Clin. Lab. Sci. 2022, 59, 156. 34851806 10.1080/10408363.2021.1997898

[232] Z. Zhu , L. Garcia-Gancedo , A. J. Flewitt , H. Xie , F. Moussy , W. I. Milne , Sensors 2012, 12, 5996. 22778628 10.3390/s120505996 PMC3386727

[233] Z. Li , W. Zhang , F. Xing , Int. J. Mol. Sci. 2019, 20, 2461. 31109057 10.3390/ijms20102461 PMC6567174

[234] R. K. Singh , N. Nalajala , T. Kar , A. Schechter , in Surface Engineering of Graphene (Eds: S. Sahoo , S. K. Tiwari , G. C. Nayak ), Springer, Berlin, Germany 2019, p. 139.

[235] I. H. Cho , D. H. Kim , S. Park , Biomater. Res. 2020, 24, 6. 32042441 10.1186/s40824-019-0181-y PMC7001310

[236] X. Chen , X. Jia , J. Han , J. Ma , Z. Ma , Biosens. Bioelectron. 2013, 50, 356. 23891798 10.1016/j.bios.2013.06.054

[237] L. Suresh , P. K. Brahman , K. R. Reddy , J. S. Bondili , Enzyme Microb. Technol. 2018, 112, 43. 29499779 10.1016/j.enzmictec.2017.10.009

[238] M. Wu , Y. Yang , K. Cao , C. Zhao , X. Qiao , C. Hong , Bioelectrochemistry 2020, 132, 107434. 31821901 10.1016/j.bioelechem.2019.107434

[239] A. Benvidi , A. D. Firouzabadi , S. M. Moshtaghiun , M. Mazloum-Ardakani , M. D. Tezerjani , Anal. Biochem. 2015, 484, 24. 25988596 10.1016/j.ab.2015.05.009

[240] Y. Bai , T. Xu , X. Zhang , Micromachines 2020, 11, 60. 31947894 10.3390/mi11010060 PMC7019259

[241] R. Salahandish , A. Ghaffarinejad , E. Omidinia , H. Zargartalebi , A. K. Majidzadeh , S. M. Naghib , A. Sanati-Nezhad , Biosens. Bioelectron. 2018, 120, 129. 30172235 10.1016/j.bios.2018.08.025

[242] T. Wei , W. Tu , B. Zhao , Y. Lan , J. Bao , Z. Dai , Sci. Rep. 2014, 4, 3982. 24496270 10.1038/srep03982 PMC3913935

[243] M. Choudhary , K. Arora , in Biosensor Based Advanced Cancer Diagnostics: From Lab to Clinics (Eds: R. Khan , A. Parihar , S. K. Sanghi ), Academic Press 2022, p. 123.

[244] M. Sharafeldin , G. W. Bishop , S. Bhakta , A. El-Sawy , S. L. Suib , J. F. Rusling , Biosens. Bioelectron. 2017, 91, 359. 28056439 10.1016/j.bios.2016.12.052 PMC5323322

[245] S. S. Low , Z. Chen , Y. Li , Y. Lu , Q. Liu , TrAc, Trends Anal. Chem. 2021, 145, 116454.

[246] H. Yin , Y. Zhou , B. Li , X. Li , Z. Yang , S. Ai , X. Zhang , Sens. Actuators B 2016, 222, 1119.

[247] S. Cajigas , J. Orozco , Molecules 2020, 25, 3542. 32756410 10.3390/molecules25153542 PMC7436128

[248] F. Ghorbani , H. Abbaszadeh , J. E. N. Dolatabadi , L. Aghebati-Maleki , M. Yousefi , Biosens. Bioelectron. 2019, 142, 111484. 31284103 10.1016/j.bios.2019.111484

[249] S. Dehghani , R. Nosrati , M. Yousefi , A. Nezami , F. Soltani , S. M. Taghdisi , K. Abnous , M. Alibolandi , M. Ramezani , Biosens. Bioelectron. 2018, 110, 23. 29579646 10.1016/j.bios.2018.03.037

[250] S. R. Yan , M. M. Foroughi , M. Safaei , S. Jahani , N. Ebrahimpour , F. Borhani , N. R. Z. Baravati , Z. Aramesh-Boroujeni , L. K. Foong , Int. J. Biol. Macromol. 2020, 155, 184. 32217120 10.1016/j.ijbiomac.2020.03.173

[251] a) J. Luo , D. Liang , X. Qiu , M. Yang , Anal. Bioanal. Chem. 2019, 411, 6889; 31401668 10.1007/s00216-019-02060-1

[252] S. Vinoth , K. S. S. Devi , A. Pandikumar , TrAc-Trends Anal. Chem. 2021, 140, 116274.

[253] S. Selvarajan , A. Suganthi , M. Rajarajan , Ultrason. Sonochem. 2018, 41, 651. 29137797 10.1016/j.ultsonch.2017.10.032

[254] S. Iravani , R. S. Varma , Environ. Chem. Lett. 2020, 18, 703. 32206050 10.1007/s10311-020-00984-0 PMC7088420

[255] Q. Gao , J. Han , Z. Ma , Biosens. Bioelectron. 2013, 49, 323. 23792654 10.1016/j.bios.2013.05.048

[256] C. Gu , C. Guo , Z. Li , M. Wang , N. Zhou , L. He , Z. Zhang , M. Du , Biosens. Bioelectron. 2019, 134, 8. 30952013 10.1016/j.bios.2019.03.043

[257] R. Yuan , H. K. Li , H. He , Dalton Trans. 2021, 50, 14091. 34609402 10.1039/d1dt02360h

[258] M. Devi , Adapting 2D Nanomaterials for Advanced Applications (Eds: L. Singh , D. M. Mahapatra ), American Chemical Society, Washington, DC, USA 2020, Ch. 6.

[259] A. Camarca , A. Varriale , A. Capo , A. Pennacchio , A. Calabrese , C. Giannattasio , C. M. Almuzara , S. D’Auria , M. Staiano , Sensors 2021, 21, 906. 33572812 10.3390/s21030906 PMC7866296

[260] C. Wurth , M. Grabolle , J. Pauli , M. Spieles , U. Resch-Genger , Nat. Protoc. 2013, 8, 1535. 23868072 10.1038/nprot.2013.087

[261] P. Moroz , *Ph.D. Thesis*, Bowling Green State University, Bowling Green, OH, USA 2015.

[262] L. Stryer , D. D. Thomas , C. F. Meares , Annu. Rev. Biophys. Bioeng. 1982, 11, 203. 7049062 10.1146/annurev.bb.11.060182.001223

[263] M. Swierczewska , G. Liu , S. Lee , X. Chen , Chem. Soc. Rev. 2012, 41, 2641. 22187721 10.1039/c1cs15238f PMC3629948

[264] S. Li , Y. Li , J. Cao , J. Zhu , L. Fan , X. Li , Anal. Chem. 2014, 86, 10201. 25280346 10.1021/ac503183y

[265] M. Liu , H. Zhao , S. Chen , H. Yu , X. Quan , Environ. Sci. Technol. 2012, 46, 12567. 23113735 10.1021/es3028583

[266] S. Lutje , M. Rijpkema , W. Helfrich , W. J. Oyen , O. C. Boerman , Mol. Imaging Biol. 2014, 16, 747. 24849133 10.1007/s11307-014-0747-y

[267] X. J. Xing , X. G. Liu , H. Yue , Q. Y. Luo , H. W. Tang , D. W. Pang , Biosens. Bioelectron. 2012, 37, 61. 22613226 10.1016/j.bios.2012.04.037

[268] Y. Yim , H. Shin , S. M. Ahn , D. H. Min , Chem. Commun. 2021, 57, 9820. 10.1039/d1cc02157e 34494621

[269] J. R. Lakowicz , B. P. Maliwal , J. Biol. Chem. 1983, 258, 4794. 6833277

[270] F. Charbgoo , F. Soltani , S. M. Taghdisi , K. Abnous , M. Ramezani , TrAc Trends Anal. Chem. 2016, 85, 85.

[271] H. Zhang , H. Zhang , A. Aldalbahi , X. Zuo , C. Fan , X. Mi , Biosens. Bioelectron. 2017, 89, 96. 27459883 10.1016/j.bios.2016.07.030

[272] X. Li , X. Ding , J. Fan , Analyst 2015, 140, 7918. 26502364 10.1039/c5an01759a

[273] J. Shi , F. Tian , J. Lyu , M. Yang , J. Mater. Chem. B 2015, 3, 6989. 32262700 10.1039/c5tb00885a

[274] Y. Yang , W. Lei , Y. Xu , T. Zhou , M. Xia , Q. Hao , Mikrochim. Acta 2017, 185, 39. 29594453 10.1007/s00604-017-2533-4

[275] K. Hu , T. M. Zhong , Y. Huang , Z. F. Chen , S. L. Zhao , Mikrochim. Acta 2015, 182, 949.

[276] X. T. Liu , H. J. Zhang , Z. P. Song , L. Q. Guo , F. F. Fu , Y. N. Wu , Biosens. Bioelectron. 2019, 129, 118. 30690175 10.1016/j.bios.2019.01.032

[277] S. Hamd-Ghadareh , B. A. Hamah-Ameen , A. Salimi , F. Fathi , F. Soleimani , Microchim. Acta 2019, 186, 469. 10.1007/s00604-019-3446-1 31240482

[278] S. Hamd-Ghadareh , A. Salimi , F. Fathi , S. Bahrami , Biosens. Bioelectron. 2017, 96, 308. 28525848 10.1016/j.bios.2017.05.003

[279] F. Zhao , S. Xie , B. Li , X. Zhang , Int. J. Biol. Macromol. 2022, 201, 592. 35031315 10.1016/j.ijbiomac.2022.01.039

[280] Y. Wu , *Ph.D. Dissertation*, Oregon State University, Corvallis, OR, USA 2016, p. 152.

[281] N. A. Alarfaj , M. F. El-Tohamy , H. F. Oraby , Int. J. Mol. Sci. 2018, 19, 1162. 29641488 10.3390/ijms19041162 PMC5979385

[282] A. Kalkal , R. Pradhan , S. Kadian , G. Manik , G. Packirisamy , ACS Appl. Bio Mater. 2020, 3, 4922. 10.1021/acsabm.0c00427 35021736

[283] P. B. Luppa , L. J. Sokoll , D. W. Chan , Clin. Chim. Acta 2001, 314, 1. 11718675 10.1016/s0009-8981(01)00629-5

[284] Y. Yao , B. Yi , J. Xiao , Z. Li , in 2007 1st Int. Conf. on Bioinformatics and Biomedical Engineering, IEEE, Piscataway, NJ, USA 2007, p. 1043.

[285] S. N. Nangare , P. O. Patil , ACS Biomater. Sci. Eng. 2021, 7, 2. 33455205 10.1021/acsbiomaterials.0c01203

[286] H. H. Nguyen , J. Park , S. Kang , M. Kim , Sensors 2015, 15, 10481. 25951336 10.3390/s150510481 PMC4481982

[287] D. R. Shankaran , K. V. A. Gobi , N. Miura , Sens. Actuators, B 2007, 121, 158.

[288] D. E. Souto , J. Volpe , D. R. de Oliveira , in Tools and Trends in Bioanalytical Chemistry, (Eds: L. T. Kubota , J. Al. Fracassi da Silva , M. M. Sena , W. A. Alves ) Springer, Berlin, Germany 2022, p. 223.

[289] N. Bellassai , R. D’Agata , V. Jungbluth , G. Spoto , Front. Chem. 2019, 7, 570. 31448267 10.3389/fchem.2019.00570 PMC6695566

[290] J. K. Nayak , P. Parhi , R. Jha , Sens. Actuators, B 2015, 221, 835.

[291] J. Y. Jing , Q. Wang , W. M. Zhao , B. T. Wang , Opt. Lasers Eng. 2019, 112, 103.

[292] B. D. Gupta , R. Kant , Opt. Laser Technol. 2018, 101, 144.

[293] F. Yesilkoy , R. A. Terborg , J. Pello , A. A. Belushkin , Y. Jahani , V. Pruneri , H. Altug , Light Sci. Appl. 2018, 7, 17152. 30839537 10.1038/lsa.2017.152 PMC6060062

[294] M. M. Rahman , M. M. Rana , M. S. Rahman , M. S. Anower , M. A. Mollah , A. K. Paul , Opt. Mater. 2020, 107, 110123.

[295] O. Tagit , N. Hildebrandt , ACS Sens. 2017, 2, 31. 28722447 10.1021/acssensors.6b00625

[296] S. Singh , P. K. Singh , A. Umar , P. Lohia , H. Albargi , L. Castaneda , D. K. Dwivedi , Micromachines 2020, 11, 779. 32824184 10.3390/mi11080779 PMC7463818

[297] M. S. Rahman , M. R. Hasan , K. A. Rikta , M. S. Anower , Opt. Mater. 2018, 75, 567.

[298] J. Cao , M. H. Tu , T. Sun , K. T. V. Grattan , Sens. Actuators, B 2013, 181, 611.

[299] J. Homola , S. S. Yee , G. Gauglitz , Sens. Actuators B 1999, 54, 3.

[300] J. Xu , P. Kvasnicka , M. Idso , R. W. Jordan , H. Gong , J. Homola , Q. Yu , Opt. Express 2011, 19, 20493. 21997057 10.1364/OE.19.020493

[301] X. Guo , J. Biophotonics 2012, 5, 483. 22467335 10.1002/jbio.201200015

[302] M. S. Rahman , M. S. Anower , L. B. Bashar , K. A. Rikta , Sens. Bio-Sens. Res. 2017, 16, 41.

[303] C.-W. Lin , C.-C. Chang , in Biosensors and Cancer (Eds: V. R. Preedy , V. Patel ), CRC Press, Boca Raton, FL, USA 2012, p. 17.

[304] M. Yang , X. Yi , J. Wang , F. Zhou , Analyst 2014, 139, 1814. 24600683 10.1039/c3an02065g

[305] Y. V. Stebunov , O. A. Aftenieva , A. V. Arsenin , V. S. Volkov , ACS Appl. Mater. Interfaces 2015, 7, 21727. 26358000 10.1021/acsami.5b04427

[306] N. F. Chiu , C. T. Kuo , C. Y. Chen , Int. J. Nanomed. 2019, 14, 4833. 10.2147/IJN.S208292 PMC6613200 31308661

[307] N. F. Chiu , T. L. Lin , C. T. Kuo , Sens. Actuators, B 2018, 265, 264.

[308] Q. Li , Q. Wang , X. Yang , K. Wang , H. Zhang , W. Nie , Talanta 2017, 174, 521. 28738618 10.1016/j.talanta.2017.06.048

[309] F. Duan , S. Zhang , L. Yang , Z. Zhang , L. He , M. Wang , Anal. Chim. Acta 2018, 1036, 121. 30253822 10.1016/j.aca.2018.06.070

[310] F. Farshchi , M. Hasanzadeh , Biomed. Pharmacother. 2020, 132, 110878. 33113419 10.1016/j.biopha.2020.110878

[311] P. Prakrankamanant , J. Med. Assoc. Thailand 2014, 97, 56. 24851566

[312] A. Roointan , T. A. Mir , S. I Wani , R. M. Ur , K. K. Hussain , B. Ahmed , S. Abrahim , A. Savardashtaki , G. Gandomani , M. Gandomani , R. Chinnappan , M. H. Akhtar , J. Pharm. Biomed. Anal. 2019, 164, 93. 30366148 10.1016/j.jpba.2018.10.017

[313] J. Kwak , S. S. Lee , Nanotechnol. 2019, 30, 445502. 10.1088/1361-6528/ab36c9 31362281

[314] D. A. Buttry , M. D. Ward , Chem. Rev. 1992, 92, 1355.

[315] A. T. Lawal , Biosens. Bioelectron. 2019, 141, 111384. 31195196 10.1016/j.bios.2019.111384

[316] M. Rodahl , F. Hook , B. Kasemo , Anal. Chem. 1996, 68, 2219. 21619308 10.1021/ac951203m

[317] H. J. Lim , T. Saha , B. T. Tey , W. S. Tan , C. W. Ooi , Biosens. Bioelectron. 2020, 168, 112513. 32889395 10.1016/j.bios.2020.112513 PMC7443316

[318] D. Chronaki , D. I. Stratiotis , A. Tsortos , E. Anastasiadou , E. Gizeli , Sens. Bio-Sens. Res. 2016, 11, 99.

[319] P. J. Jandas , J. Luo , A. Quan , C. Li , C. Fu , Y. Q. Fu , RSC Adv. 2020, 10, 4118. 35492675 10.1039/c9ra09963h PMC9049092

[320] S. P. Usha , H. Manoharan , R. Deshmukh , R. Alvarez-Diduk , E. Calucho , V. V. R. Sai , A. Merkoci , Chem. Soc. Rev. 2021, 50, 13012. 34673860 10.1039/d1cs00137j

[321] N. Atar , M. L. Yola , Anal. Chim. Acta 2021, 1148, 338202. 33516376 10.1016/j.aca.2021.338202

[322] L. Zhao , H. Han , Z. Ma , Biosens. Bioelectron. 2018, 101, 304. 29107882 10.1016/j.bios.2017.10.041

[323] W. Gao , W. Wang , S. Yao , S. Wu , H. Zhang , J. Zhang , F. Jing , H. Mao , Q. Jin , H. Cong , C. Jia , G. Zhang , J. Zhao , Anal. Chim. Acta 2017, 958, 77. 28110687 10.1016/j.aca.2016.12.016

[324] A. V. Baskar , M. R. Benzigar , S. N. Talapaneni , G. Singh , A. S. Karakoti , J. Yi , A. a. H. Al-Muhtaseb , K. Ariga , P. M. Ajayan , A. Vinu , Adv. Funct. Mater. 2021, 32, 2106924.

[325] A. V. Baskar , A. M. Ruban , J. M. Davidraj , G. Singh , A. a. H. Al-Muhtaseb , J. M. Lee , J. Yi , A. Vinu, Bull. Chem. Soc. Jpn. 2021, 94, 133. 10.1166/jnn.2021.19141 33404411

[326] M. R. Benzigar , S. Joseph , G. Saianand , A. I. Gopalan , S. Sarkar , S. Srinivasan , D. H. Park , S. Kim , S. N. Talapaneni , K. Ramadass , A. Vinu , Microporous Mesoporous Mater. 2019, 285, 21.

[327] M. R. Benzigar , S. Joseph , H. Ilbeygi , D. H. Park , S. Sarkar , G. Chandra , S. Umapathy , S. Srinivasan , S. N. Talapaneni , A. Vinu , Angew. Chem., Int. Ed. 2018, 57, 569. 10.1002/anie.201710888 29114988

[328] M. R. Benzigar , S. Joseph , A. V. Baskar , D. H. Park , G. Chandra , S. Umapathy , S. N. Talapaneni , A. Vinu , Adv. Funct. Mater. 2018, 28, 1803701.

[329] S. Afreen , K. Muthoosamy , S. Manickam , U. Hashim , Biosens. Bioelectron. 2015, 63, 354. 25125029 10.1016/j.bios.2014.07.044

[330] M. Gaur , C. Misra , A. B. Yadav , S. Swaroop , F. O. Maolmhuaidh , M. Bechelany , A. Barhoum , Materials 2021, 14, 5978. 34683568 10.3390/ma14205978 PMC8538389

[331] S. Pilehvar , K. De Wael , in Nanocarbons for Electroanalysis (Eds: S. Szunerits , R. Boukherroub , A. Downard , J-J. Zhu ), John Wiley & Sons Ltd., Hoboken, NJ, USA 2017, Ch. 7.

[332] X. Guan , P. Kumar , Z. Li , T. K. A. Tran , S. Chahal , Z. Lei , C. Y. Huang , C. H. Lin , J. K. Huang , L. Hu , Y. C. Chang , L. Wang , J. S. J. Britto , L. Panneerselvan , D. Chu , T. Wu , A. Karakoti , J. Yi , A. Vinu , Adv. Sci. 2023, 10, 2205809. 10.1002/advs.202205809 PMC9982542 36698305

[333] H. S. Gujral , G. Singh , A. V. Baskar , X. Guan , X. Geng , A. V. Kotkondawar , S. Rayalu , P. Kumar , A. Karakoti , A. Vinu , Sci. Technol. Adv. Mater. 2022, 23, 76. 35309252 10.1080/14686996.2022.2029686 PMC8928826

[334] D. Wu , Y. Liu , Y. Wang , L. Hu , H. Ma , G. Wang , Q. Wei , Sci. Rep. 2016, 6, 20511. 26842737 10.1038/srep20511 PMC4740800

[335] J. A. Jaleel , K. Pramod , J. Controlled Release 2018, 269, 302. 10.1016/j.jconrel.2017.11.027 29170139

[336] X. W. Zhao , J. L. Zhang , L. H. Shi , M. Xian , C. Dong , S. M. Shuang , RSC Adv. 2017, 7, 42159.

[337] S. Lu , S. Guo , P. Xu , X. Li , Y. Zhao , W. Gu , M. Xue , Int. J. Nanomed. 2016, 11, 6325. 10.2147/IJN.S119252 PMC5135288 27932880

[338] L. Li , R. Zhang , C. Lu , J. Sun , L. Wang , B. Qu , T. Li , Y. Liu , S. Li , J. Mater. Chem. B 2017, 5, 7328. 32264182 10.1039/c7tb00634a

[339] A. A. Myint , W. K. Rhim , J. M. Nam , J. Kim , Y. W. Lee , J. Ind. Eng. Chem. 2018, 66, 387.

[340] H. Ding , L. W. Cheng , Y. Y. Ma , J. L. Kong , H. M. Xiong , New J. Chem. 2013, 37, 2515.

[341] Y. Zhuo , H. Miao , D. Zhong , S. S. Zhu , X. M. Yang , Mater. Lett. 2015, 139, 197.

[342] L. Ge , H. Yu , H. Ren , B. Shi , Q. Guo , W. Gao , Z. Li , J. Li , J. Mater. Sci. 2017, 52, 9979.

[343] V. N. Mehta , S. Jha , R. K. Singhal , S. K. Kailasa , New J. Chem. 2014, 38, 6152.

[344] M. Xue , L. Zhang , Z. Zhan , M. Zou , Y. Huang , S. Zhao , Talanta 2016, 150, 324. 26838415 10.1016/j.talanta.2015.12.024

[345] K. M. Tripathi , T. S. Tran , T. T. Tung , D. Losic , T. Kim , J. Nanomater. 2017, 7029731.

[346] H. D. Jang , S. K. Kim , H. Chang , J. W. Choi , Biosens. Bioelectron. 2015, 63, 546. 25150936 10.1016/j.bios.2014.08.008

[347] L. H. Pan , S. H. Kuo , T. Y. Lin , C. W. Lin , P. Y. Fang , H. W. Yang , Biosens. Bioelectron. 2017, 89, 598. 26868935 10.1016/j.bios.2016.01.077

[348] M. Yan , G. Sun , F. Liu , J. Lu , J. Yu , X. Song , Anal. Chim. Acta 2013, 798, 33. 24070481 10.1016/j.aca.2013.08.046

[349] Z. Zhong , W. Wu , D. Wang , D. Wang , J. Shan , Y. Qing , Z. Zhang , Biosens. Bioelectron. 2010, 25, 2379. 20353889 10.1016/j.bios.2010.03.009

[350] H. Yin , Y. Zhou , C. Chen , L. Zhu , S. Ai , Analyst 2012, 137, 1389. 22311172 10.1039/c2an16098f

[351] Y. Wang , H. Xu , J. Luo , J. Liu , L. Wang , Y. Fan , S. Yan , Y. Yang , X. Cai , Biosens. Bioelectron. 2016, 83, 319. 27132007 10.1016/j.bios.2016.04.062

[352] C. Pothipor , N. Wiriyakun , T. Putnin , A. Ngamaroonchote , J. Jakmunee , K. Ounnunkad , R. Laocharoensuk , N. Aroonyadet , Sens. Actuators, B 2019, 296, 126657.

[353] S. Kumar , J. G. Sharma , S. Maji , B. D. Malhotra , Biosens. Bioelectron. 2016, 78, 497. 26657594 10.1016/j.bios.2015.11.084

[354] Q. Zhu , Y. Chai , Y. Zhuo , R. Yuan , Biosens. Bioelectron. 2015, 68, 42. 25562732 10.1016/j.bios.2014.12.023

[355] B. Jin , P. Wang , H. Mao , B. Hu , H. Zhang , Z. Cheng , Z. Wu , X. Bian , C. Jia , F. Jing , Q. Jin , J. Zhao , Biosens. Bioelectron. 2014, 55, 464. 24462797 10.1016/j.bios.2013.12.025

[356] X. Jia , X. Chen , J. Han , J. Ma , Z. Ma , Biosens. Bioelectron. 2014, 53, 65. 24113435 10.1016/j.bios.2013.09.021

[357] S. Kumar , J. G. Sharma , S. Maji , B. D. Malhotra , RSC Adv. 2016, 6, 77037.

[358] J. Liu , J. Wang , T. Wang , D. Li , F. Xi , J. Wang , E. Wang , Biosens. Bioelectron. 2015, 65, 281. 25461170 10.1016/j.bios.2014.10.016

[359] Y. X. Yang , M. Jiang , K. H. Cao , M. Wu , C. L. Zhao , H. L. Li , C. L. Hong , Microchem. J. 2019, 151, 104223.

[360] S. Augustine , P. Kumar , B. D. Malhotra , ACS Appl. Bio Mater. 2019, 2, 5366. 10.1021/acsabm.9b00659 35021536

[361] W. Wen , J. Y. Huang , T. Bao , J. Zhou , H. X. Xia , X. H. Zhang , S. F. Wang , Y. D. Zhao , Biosens. Bioelectron. 2016, 83, 142. 27111123 10.1016/j.bios.2016.04.039

[362] Q. F. Li , D. P. Tang , F. M. Lou , X. M. Yang , G. N. Chen , ChemElectroChem 2014, 1, 441.

[363] T. Xu , N. Liu , J. Yuan , Z. Ma , Biosens. Bioelectron. 2015, 70, 161. 25814405 10.1016/j.bios.2015.03.036

[364] A. Tabasi , A. Noorbakhsh , E. Sharifi , Biosens. Bioelectron. 2017, 95, 117. 28433858 10.1016/j.bios.2017.04.020

[365] M. Srivastava , N. R. Nirala , S. K. Srivastava , R. Prakash , Sci. Rep. 2018, 8, 1923. 29386538 10.1038/s41598-018-19733-z PMC5792442

[366] Y. Zeng , J. Bao , Y. Zhao , D. Huo , M. Chen , M. Yang , H. Fa , C. Hou , Talanta 2018, 178, 122. 29136801 10.1016/j.talanta.2017.09.020

[367] H. Jia , Q. Tian , J. Xu , L. Lu , X. Ma , Y. Yu , Mikrochim. Acta 2018, 185, 517. 30362031 10.1007/s00604-018-3056-3

[368] J. Shi , J. Lyu , F. Tian , M. Yang , Biosens. Bioelectron. 2017, 93, 182. 27614683 10.1016/j.bios.2016.09.012

[369] Z. Wang , S. Wu , L. C. Ciacchi , G. Wei , Analyst 2018, 143, 5074. 30280724 10.1039/c8an01266k

[370] S. Mohammadi , A. Salimi , S. Hamd-Ghadareh , F. Fathi , F. Soleimani , Anal. Biochem. 2018, 557, 18. 29908158 10.1016/j.ab.2018.06.008

[371] M. Mahani , Z. Mousapour , F. Divsar , A. Nomani , H. Ju , Mikrochim. Acta 2019, 186, 132. 30707293 10.1007/s00604-019-3233-z

[372] Y. Y. Wu , P. Wei , S. Pengpumkiat , E. A. Schumacher , V. T. Remcho , Anal. Methods 2016, 8, 5398.

[373] D. Zhong , K. Yang , Y. Wang , X. Yang , Talanta 2017, 175, 217. 28841982 10.1016/j.talanta.2017.07.035

[374] N. Ma , W. T. Jiang , T. Li , Z. Q. Zhang , H. Z. Qi , M. H. Yang , Microchim. Acta 2015, 182, 443.

[375] X. Fang , X. Q. Li , H. Wang , X. M. Wu , G. L. Wang , Sens. Actuators, B 2018, 257, 620.

[376] O. Zagorodko , J. Spadavecchia , A. Y. Serrano , I. Larroulet , A. Pesquera , A. Zurutuza , R. Boukherroub , S. Szunerits , Anal. Chem. 2014, 86, 11211. 25341125 10.1021/ac502705n

